# Activated leukocyte cell adhesion molecule expression correlates with the WNT subgroup in medulloblastoma and is involved in regulating tumor cell proliferation and invasion

**DOI:** 10.1371/journal.pone.0243272

**Published:** 2020-12-03

**Authors:** Takamune Achiha, Noriyuki Kijima, Yoshinori Kodama, Naoki Kagawa, Manabu Kinoshita, Yasunori Fujimoto, Masahiro Nonaka, Junya Fukai, Akihiro Inoue, Namiko Nishida, Takumi Yamanaka, Atsuko Harada, Kanji Mori, Naohiro Tsuyuguchi, Takehiro Uda, Kenichi Ishibashi, Yusuke Tomogane, Daisuke Sakamoto, Tomoko Shofuda, Ema Yoshioka, Daisuke Kanematsu, Masayuki Mano, Betty Luu, Michael D. Taylor, Yonehiro Kanemura, Haruhiko Kishima

**Affiliations:** 1 Department of Neurosurgery, Osaka University Graduate School of Medicine, Osaka, Japan; 2 Kansai Molecular Diagnosis Network for Central Nervous System Tumors, Osaka, Japan; 3 Department of Neurosurgery, Osaka National Hospital, National Hospital Organization, Osaka, Japan; 4 Department of Pathology and Applied Neurobiology, Kyoto Prefectural University of Medicine, Kyoto, Japan; 5 Department of Central Laboratory and Surgical Pathology, Osaka National Hospital, National Hospital Organization, Osaka, Japan; 6 Department of Neurosurgery, Kansai Medical University, Hirakata, Japan; 7 Department of Neurological Surgery, Wakayama Medical University School of Medicine, Wakayama, Japan; 8 Department of Neurosurgery, Ehime University School of Medicine, Ehime, Japan; 9 Department of Neurosurgery, Tazuke Kofukai Foundation, Medical Research Institute, Kitano Hospital, Osaka, Japan; 10 Department of Neurosurgery, Kyoto Prefectural University of Medicine, Kyoto, Japan; 11 Department of Pediatric Neurosurgery, Takatsuki General Hospital, Osaka, Japan; 12 Department of Neurosurgery, Kansai Rosai Hospital, Amagasaki, Japan; 13 Department of Neurosurgery, Kindai University Faculty of Medicine, Sayama, Japan; 14 Department of Neurosurgery, Osaka City University Graduate School of Medicine, Osaka, Japan; 15 Department of Neurosurgery, Osaka City General Hospital, Osaka, Japan; 16 Department of Neurosurgery, Hyogo College of Medicine, Hyogo, Japan; 17 Department of Biomedical Research and Innovation Research, Institute for Clinical Research, Osaka National Hospital, National Hospital Organization, Osaka, Japan; 18 Developmental and Stem Cell Biology Program, The Hospital for Sick Children, Toronto, Ontario, Canada; 19 The Arthur and Sonia Labatt Brain Tumour Research Centre, The Hospital for Sick Children, Toronto, Ontario, Canada; 20 Division of Neurosurgery, The Hospital for Sick Children, Toronto, Ontario, Canada; 21 Department of Surgery, Department of Laboratory Medicine and Pathobiology, and Department of Medical Biophysics, University of Toronto, Toronto, Ontario, Canada; University of Navarra, SPAIN

## Abstract

Cluster of differentiation (CD) 166 or activated leukocyte cell adhesion molecule (ALCAM) is a transmembrane molecule known to be an intercellular adhesion factor. The expression and function of ALCAM in medulloblastoma (MB), a pediatric brain tumor with highly advanced molecular genetics, remains unclear. Therefore, this study aimed to clarify the significance and functional role of ALCAM expression in MB. ALCAM expression in 45 patients with MB was evaluated by immunohistochemical analysis of formalin-fixed paraffin-embedded clinical specimens and the relationship between ALCAM expression and pathological type/molecular subgroup, such as WNT, SHH, Group 3, and Group 4, was examined. Eight ALCAM positive (18%), seven partially positive (16%), and 30 negative (67%) cases were detected. All seven cases of the WNT molecular subgroup were ALCAM positive and ALCAM expression strongly correlated with this subgroup (*P* < 0.0001). In addition, functional studies using MB cell lines revealed ALCAM expression affected proliferation and migration as a positive regulator *in vitro*. However, ALCAM silencing did not affect survival or the formation of leptomeningeal dissemination in an orthotopic mouse model, but did induce a malignant phenotype with increased tumor cell invasion at the dissemination sites (P = 0.0029). In conclusion, our results revealed that ALCAM exhibited highly specific expression in the WNT subgroup of MB. Furthermore, we demonstrated that the cell kinetics of MB cell lines can be altered by the expression of ALCAM.

## Introduction

Medulloblastoma (MB) is the most common pediatric malignant brain tumor of the cerebellum. Histologically, MB is an embryonal tumor that may differentiate into cells of neural lineages and is classified as a grade IV tumor by the World Health Organization (WHO). There are several MB histological subtypes, including classic, desmoplastic/nodular, MB with extensive nodularity (MBEN), and large cell/anaplastic. Patients with MB are clinically stratified into average or high-risk groups according to their age, metastatic status, and the presence of residual tumor following resection [1]. However, these classifications do not account for MB heterogeneity. Recently, integrated genomic analysis of MB showed that MB consists of at least four distinct molecular subgroups, WNT, SHH, Group 3, and Group 4 [2–4]. This molecular classification of MB reflects distinct demographics and clinical features, including prognosis, transcriptomes, and genetics [2–5], and have been incorporated into the WHO classification of tumors of the central nervous system as revised in 2016 [6]. This new classification also provides for additional clinical risk stratification [5, 7]. However, a further understanding of disease based on the molecular subgroups and the subsequent development of treatment strategies is necessary.

Activated leukocyte cell adhesion molecule (ALCAM) is a transmembrane glycoprotein that belongs to the immunoglobulin superfamily. ALCAM has been identified in a wide variety of tissues and cells, such as selected epithelia, lymphoid and myeloid cells, fibroblasts, neurons, and hepatocytes and is also referred to as CD166, CD6 ligand, MEMD, SB10 antigen, and HCA [8]. ALCAM is involved in cell-cell adhesion, either by homophilic (ALCAM-ALCAM) or heterophilic (ALCAM-CD6) interaction, and is also involved in organ development, neurogenesis, hematopoiesis, and immune responses [8]. In cancer, ALCAM expression is a prognostic marker for various tumor types [9]. For example, membranous ALCAM expression and ALCAM overexpression are independent markers of poor prognosis in colorectal carcinoma and pancreatic cancer, respectively [10, 11]. Contrarily, decreased ALCAM expression in breast cancer and loss of ALCAM membrane expression in ovarian cancer have been correlated with poor prognosis [12, 13]. Therefore, the impact of ALCAM expression on prognosis seems to depend on the cancer type, and in some types of cancer, membranous versus cytoplasmic ALCAM expression should also be considered [9]. *In vitro* and *in vivo* studies have demonstrated that ALCAM is involved in migration, invasion, and stemness in several cancers [14–19]. However, the functional significance of ALCAM in cancer is not consistent, with the differences depending on both the cancer type and tumor microenvironment.

The expression of ALCAM in MB has been previously reported, but its relevance to the molecular subgroups or histological classification has not been examined [20]. Furthermore, no study has analyzed the functional role of ALCAM in MB. In the current study, we retrospectively evaluated ALCAM expression in samples from patients with MB and examined the correlation between ALCAM expression and the MB molecular subgroups and histological subtypes. In addition, we investigated the functional role of ALCAM in MB using *in vitro* assays and an *in vivo* orthotopic mouse model.

## Materials and methods

### Clinical samples and patient characteristics

We retrospectively recruited sample cases that a) had available specimens surgically removed between 1996 and 2020 and b) were diagnosed with MB at the original treating institute. All cases were then centrally reviewed by a senior board-certified neuropathologist (Y.K.) for inclusion in the study. Forty-five case specimens of MB and their clinicopathologic information were obtained from 11 collaborating institutions (Osaka University Graduate School of Medicine, Osaka National Hospital, Kansai Medical University, Wakayama Medical University School of Medicine, Ehime University School of Medicine, Kitano Hospital, Takatsuki General Hospital, Kansai Rosai Hospital, Osaka City University Graduate School of Medicine, Osaka City General Hospital, and Hyogo College of Medicine) in the Kansai Molecular Diagnosis Network for CNS Tumors [21]. Approval of the study was obtained from the Institutional Review Boards (IRBs) of Osaka University Graduate School of Medicine (approval number: 13244), Osaka National Hospital (approval number: 713), and all the collaborative institutes. For all cases, either written informed consent was obtained or its requirement was waived by the IRB with a public announcement on the institution website. Immunohistochemistry and data analysis were performed at Osaka University Graduate School of Medicine and genetic analysis was performed at Osaka National Hospital and The Hospital for Sick Children. MB was histologically classified based on hematoxylin-eosin (HE) and reticulin silver staining as classic, desmoplastic/nodular, MBEN, or large cell/anaplastic subtype according to the 2016 WHO classification.

### Immunohistochemistry

Six-micrometer sections of formalin-fixed paraffin-embedded (FFPE) tissues were used for immunohistochemistry. Heat-induced antigen retrieval was performed using a pressure cooker in 0.01 M citrate buffer (pH 6.0) for 10 min. Sections were incubated with a primary antibody against CD166/ALCAM [EPR2759(2); Abcam, Cambridge, MA, USA; 1:100 dilution] and β-catenin (BD Bioscience, San Jose, CA, USA; 1:100 dilution) at 4 °C overnight. Histofine Simple Stain MAX-PO (Nichirei, Tokyo, Japan) was used as a secondary antibody. The antibody complexes were visualized using the Dako Liquid DAB + Substrate Chromogen System (Dako, Carpinteria, CA, USA) and the sections were then counterstained with hematoxylin.

To identify tumor cells in the orthotopic mouse model, a primary antibody to human STEM121 (Takara Bio Inc., Shiga, Japan; 1:1,000 dilution) was used with POD Conjugate Set Anti Mouse, For Mouse Tissue reagent (Takara Bio Inc.).

When we analyzed ALCAM expression, we also evaluated whether the membrane or cytoplasm of the tumor cells were stained. The immunostaining of ALCAM was evaluated as the proportion of ALCAM-positive tumor cells in a representative area of tumor in the section. The cutoff values for the subdivision of the ALCAM staining was set at < 1% for negative staining, 1–25% for partially positive staining, and > 25% for positive staining. β-catenin immunostaining of tumor cells was considered positive only in cases of nuclear staining.

For ALCAM immunohistochemical staining, the identification of optimal cutoff points for the proportion of ALCAM-positive tumor cells in the WNT molecular subgroup was evaluated using receiver operating characteristic (ROC) curves and assessment of the area under the ROC curve (AUC).

### Molecular subgrouping and genetic analysis

All samples were analyzed with molecular diagnostic techniques using the nanoString nCounter system (NanoString Technologies Inc., Seattle, WA, USA) [3, 4, 22] and by Sanger sequencing. The *CTNNB1* mutation hotspot region in exon 3 was amplified and sequenced using forward primer 5’-TGGAACCAGACAGAAAAGCG-3’ and reverse primer 5’-ACAGGACTTGGGAGGTATCC-3’. Cases classified by the nanoString nCounter analysis or that presented with the *CTNNB1* mutation and nuclear staining of β-catenin were defined as WNT subtype.

### Quantitative PCR (qPCR)

Total RNA was extracted from cultured cells and clinical samples using an RNeasy Mini Kit (Qiagen, Valencia, CA, USA) or QIAzol Lysis Reagent (Qiagen) and reverse transcribed using PrimeScript RT Master Mix (Takara Bio Inc.) following the manufacturers’ protocols. qPCR was performed using TB Green Premix Ex Taq II (Takara Bio Inc.) and the Applied Biosystems ViiA7 Real-Time PCR System (Thermo Fisher Scientific Inc., Waltham, MA, USA). To measure *ALCAM* expression, the following primer pair was used: 5’-TCCTGCCGTCTGCTCTTCT-3’ (forward) and 5’-TTCTGAGGTACGTCAAGTCGG-3’ (reverse) [19]. As an internal reference for normalization, *ACTB* expression was measured using the following primer pair: 5’-CACCAACTGGGACGACAT-3’ (forward) and 5’-ACAGCCTGGATAGCAACG-3’ (reverse). Expression was measured relative to Human Brain, Cerebellum Total RNA (Takara Bio) and relative quantification analyses were performed using the ΔΔCT method [23].

### R2: Genomics analysis and visualization platform dataset analysis

To validate *ALCAM* expression in MB, the Cavalli-763 MB dataset [24] from the R2: Genomics Analysis and Visualization Platform (http://r2.amc.nl) was used. *ALCAM* gene expression, patient age, histological variant, and molecular subgroup data were extracted from the dataset.

### Cell lines

Four established human MB cell lines were used in this study, Daoy, D341, ONS-76, and D283. Daoy, D341, and D283 cells were obtained from the American Type Culture Collection (ATCC, Manassas, VA, USA). ONS-76 was purchased from the Japanese Collection of Research Bioresources Cell Bank. Daoy and ONS-76 were cultured as adherent cells and D341 and D283 were cultured as cell suspensions. Daoy cells were cultured in Dulbecco’s modified Eagle’s medium (DMEM) supplemented with 10% fetal bovine serum (FBS); D341 cells were cultured in Minimum Essential Medium (MEM) supplemented with 20% FBS and 1% Non-Essential Amino Acids Solution (Gibco, Grand Island, NY, USA); ONS-76 cells were cultured in RPMI-1640 medium supplemented with 10% FBS, and D283 cells were cultured in MEM supplemented with 10% FBS. Cultured cells were maintained at 37°C in a 5% CO_2_ atmosphere.

### Flow cytometric analysis

Cells from subconfluent monolayer cultures were suspended in phosphate-buffered saline (PBS) and incubated with phycoerythrin (PE)-conjugated mouse anti-human CD166/ALCAM antibody (3A6; BD Bioscience). The labeled cells were then analyzed on a FACS Aria III flow cytometer (BD Bioscience) according to the manufacturer’s instructions. Analysis of the flow cytometry data was performed using FlowJo, version 10 software (BD Bioscience). ALCAM fluorescence intensity is reported as the intensity ratio (IR) obtained as follows: IR = ALCAM mean fluorescence intensity / negative control mean fluorescence intensity.

### Knockdown of *ALCAM* expression

To generate cell lines in which *ALCAM* expression had been stably knocked down, we used the MISSION^®^ RNAi system and lentiviral pLKO-puro vector (Sigma Aldrich, St Louis, MO, USA). The sequences of the shRNAs were as follows: shALCAM1, 5’-CCGGCAGCCATGATAATAGGTCATACTCGAGTATGACCTATTATCATGGCTGTTTTTG-3’ and shALCAM2, 5’-CCGGCTTCGATCTAGCCCGTCATTTCTCGAGAAATGACGGGCTAGATCGAAGTTTTTG-3’. Non-target shRNA Control (Sigma Aldrich) was used as negative control shRNA. Lentivirus was produced by transfection of the lentiviral vector and the Lentiviral Packaging Mix (Sigma Aldrich) in 293T cells. Daoy and ONS-76 cells were then infected with the lentivirus expressing the shRNAs. Knockdown of *ALCAM* was confirmed by flow cytometry and qPCR after puromycin selection. Before performing the *in vitro* and *in vivo* assays, the 10–15% cells with lower *ALCAM* expression were sorted from the heterogeneous Daoy cell population in which *ALCAM* had been knocked down (expressing different levels of the protein) by FACS using PE-conjugated mouse anti-human ALCAM antibody.

### *ALCAM* overexpression

The expression vector *ALCAM* cDNA ORF clone pCMV3-C-GFPSpark (Sino Biological, Beijing, China) was used for ALCAM overexpression. D341 cells were transfected with the ALCAM cDNA plasmid using a lipofection method and Sinofection (Sino Biological). pCMV3-C-GFPSpark Control Vector (Sino Biological) was used as negative control. Overexpression of *ALCAM* was confirmed by qPCR after hygromycin B selection.

### Cell proliferation assays

For cell proliferation assays, Daoy and ONS-76 cells were seeded into 6-well plates (2.0 × 10^4^ cells per well). After incubation for 48 h and 96 h, live cells were counted using a OneCell Counter (BMS, Tokyo, Japan). For D341 cell proliferation assays, cells were seeded into 12-well plates (2.0 x 10^5^ cells in 2 ml of medium per well) and live cells counted after incubation for 96 h and 192 h.

### Wound healing assays

For wound healing assays, 1.2 × 10^5^ Daoy cells or 1.6 × 10^5^ ONS-76 cells were seeded into 12-well plates and incubated until confluency (24 h). A wound was made in the cell monolayer by scraping with a CELL Scratcher (AGC Techno Glass Co., Shizuoka, Japan). The wound gap width was measured at 0, 24, and 48 h for the Daoy cells and 0, 12, and 24 h for the ONS-76 cells using a Nikon TMS inverted microscope (Nikon, Tokyo, Japan) and WRAYCAM camera (WRAYMER INC., Osaka, Japan). Images were analyzed using Photoshop CC (Adobe Systems Co., San Jose, CA, USA).

### Orthotopic mouse model

Male NOD/Shi-scid, IL-2RγKO Jic mice (7–8 weeks; In-Vivo Science Inc., Tokyo, Japan) were used for xenograft implantation. All animal experiments were performed with the approval of the Institutional Animal Care and Use Committee at the Osaka University Medical School (approval number: 29–041). All procedures involving animals were performed according to the animal use guidelines of the Animal Experiment Committee of Osaka University. The mice were anesthetized via intraperitoneal injection with three mixed sedatives (midazolam, butorphanol tartrate, and buprenorphine) and 2 × 10^5^ stably transduced Daoy shControl or Daoy shALCAM1 cells in 2 μL of PBS were injected (n = 11 per group) through a burr hole into the right cerebellar hemisphere using a stereotactic injector (Stoelting, Wood Dale, IL). The mice were monitored every 1–2 days and immediately euthanized by overdose-anesthesia when severe neurological symptoms such as weight loss, loss of mobility, and severe paralysis were observed. The brains and spinal cords of the mice were subsequently dissected and formalin-fixed for histopathological analysis. For each mouse, the brain was sliced using a Mouse Brain Slicer (Muromati Kikai Co., Tokyo, Japan) to obtain three cerebral and three cerebellar coronal sections and the spinal cord was sliced to obtain five coronal sections. All dissected tissues were stained with HE and immunostained for CD166/ALCAM. STEM121 was used as a cytoplasm marker. Leptomeningeal dissemination and invasion at dissemination sites to the cerebrum, brain stem, and spinal cord of each section were evaluated histologically. Invasion was defined as a lesion that was continuous from the disseminated lesion and infiltrated into the subpial parenchyma. The histological evaluation of each section was performed blindly by three independent observers and the scores obtained were averaged.

### Transwell assay

The invasion ability of ALCAM-depleted Daoy cells was assessed using a BioCoat Matrigel Invasion Chamber (BD Bioscience) according to the manufacturer’s instructions. Briefly, 1.0 × 10^5^ Daoy cells in serum-free DMEM were seeded onto the Matrigel insert membrane and control insert membrane. DMEM containing 10% fetal bovine serum was added to the lower chamber as a chemoattractant and the chambers were incubated for 22 h. Afterward, the cells on the lower surface of the membrane were stained with Diff-Quik (Sysmex Co., Hyogo, Japan) and counted. The invasion percentage was calculated as follows:

%Invasion=(meannumberofcellsinvadingthroughMatrigelinsertmembrane)/(meannumberofcellsmigratingthroughcontrolinsertmembrane)×100.

### Statistical analysis

All statistical analyses were performed using JMP, version 13 software (SAS Institute, Cary, NC, USA). Analysis of the relationship between ALCAM expression and the clinicopathological and molecular genetic parameters was tested using univariate analysis with the Fisher’s exact test. Comparison of *ALCAM* expression between groups in the R2: Genomics Analysis and Visualization Platform was performed using Student’s t-test and Tukey-Kramer test. For *in vitro* and *in vivo* studies, the distribution of data was verified with the Shapiro–Wilk test and parametric (One-way ANOVA and Dunnet’s post hoc test) and non-parametric (Wilcoxon rank sum test) methods were used for statistical analysis of the data. Survival data from the mouse model is presented in Kaplan-Meier plots and was analyzed with the log-rank test. Data are expressed as means ± SE. For all analyses, a *P* value < 0.05 was considered statistically significant.

## Results

### ALCAM expression correlated with the WNT molecular subgroup of MB

We investigated ALCAM expression in 45 human FFPE MB specimens using immunohistochemistry. We detected eight cases positive for ALCAM staining (18%), seven partially positive cases (16%), and 30 negative cases (67%). Interestingly, ALCAM expression showed a strong significant correlation to MB molecular subgroups (*P* < 0.0001; Table 1). All seven WNT cases and one SHH case, which histologically belongs to the MBEN subgroup, were ALCAM positive (Table 2). There was no clear correlation between ALCAM expression and the histologic variant of MB (*P* = 0.32; Table 1). Factors related to the WNT subgroup, such as nuclear β-catenin expression and *CTNNB1* mutation status, correlated with ALCAM expression (*P* < 0.0001; Table 1).

**Table 1:** Correlation between clinicopathological/molecular data and ALCAM expression.

	ALCAM positive (n = 8)	ALCAM negative (n = 30) & partially positive (n = 7)	*P* value
Age group			**0.022**
Infant (< 4 years)	1	7	
Child (4–16 years)	3	27	
Adult (> 16 yeas)	4	3	
Gender			0.19
Male	4	29	
Female	4	8	
Molecular subgroup			**< 0.0001**
WNT	7	0	
SHH	1	7	
Group 3	0	7	
Group 4	0	21	
N/A	0	2	
Histological variant			0.32
classic	7	29	
desmoplastic/nodular	0	5	
MBEN	1	0	
LCA	0	1	
*CTNNB1* status			**< 0.0001**
mutation	7	1^a^	
wild type	1	36	
Nuclear β-catenin expression			**< 0.0001**
positive	6	0	
negative	2	37	

ALCAM, activated leukocyte cell adhesion molecule; N/A, data not available; MBEN, medulloblastoma with extensive nodularity; LCA, large cell/anaplastic.

^a^This case had a *CTNNB1* silent mutation. Statistically significant findings are in bold.

**Table 2:** Overview of clinicopathological/molecular characteristics and ALCAM expression in the MB cases examined.

Case No.	Age group	molecular subgroup	Histological type	*CTNNB1* mutation status	Nuclear β-catenin expression	ALCAM expression (positive cell proportion)	ALCAM staininglocalization
MB22	adult^a^	WNT	classic	c.94G>A, p.D32N	Positive	Positive (> 75%)	Cytoplasm + partially membrane
MB29	adult^a^	WNT	classic	c.98C>T, p.S33F	Positive	Positive (> 50%)	Cytoplasm
MB32	adult^a^	WNT	classic	c.134C>T, p.S45F	Positive	Positive (> 75%)	Cytoplasm
MB34	child	WNT	classic	c.101G>A, p.G34E	Negative	Positive (> 75%)	Cytoplasm
MB43	adult	WNT	classic	c.98C>G, p.S33C	Positive	Positive (>75%)	Cytoplasm
MB9	child	WNT^b^	classic	c.95A>G, p.D32G	Positive	Positive (> 75%)	Cytoplasm + partially membrane
MB30	child	WNT^b^	classic	c.98C>T, p.S33F	Positive	Positive (> 75%)	Cytoplasm + partially membrane
MB12	child	SHH	classic	NM	Negative	Negative	N/A
MB40	adult	SHH	classic	NM	Negative	Negative	N/A
MB45	child	SHH	classic	NM	Negative	Negative	N/A
MB7	adult	SHH	desmoplastic/nodular	NM	Negative	Negative	N/A
MB25	infant	SHH	desmoplastic/nodular	NM	Negative	Negative	N/A
MB36	child	SHH	desmoplastic/nodular	NM	Negative	Negative	N/A
MB37	infant	SHH	desmoplastic/nodular	NM	Negative	Negative	N/A
MB16	infant	SHH	MBEN	NM	Negative	Positive (> 75%)	Membrane + partially cytoplasm
MB5	child	Group 3	classic	NM	Negative	Partially positive	Cytoplasm
MB13	infant	Group 3	classic	NM	Negative	Partially positive	Cytoplasm
MB11	child	Group 3	classic	NM	Negative	Negative	N/A
MB20	infant	Group 3	classic	NM	Negative	Negative	N/A
MB21	child	Group 3	classic	NM	Negative	Negative	N/A
MB35	adult	Group 3	classic	c.91C>T, p.L31L^c^	Negative	Negative	N/A
MB39	child	Group 3	classic	NM	Negative	Negative	N/A
MB3	child	Group 4	classic	NM	Negative	Partially positive	Cytoplasm
MB17	child	Group 4	classic	NM	Negative	Partially positive	Cytoplasm
MB1	child	Group 4	classic	NM	Negative	Negative	N/A
MB2	child	Group 4	classic	NM	Negative	Negative	N/A
MB6	child	Group 4	classic	NM	Negative	Negative	N/A
MB8	child	Group 4	classic	NM	Negative	Negative	N/A
MB15	child	Group 4	classic	NM	Negative	Negative	N/A
MB23	child	Group 4	classic	NM	Negative	Negative	N/A
MB24	child	Group 4	classic	NM	Negative	Negative	N/A
MB26	child	Group 4	classic	NM	Negative	Negative	N/A
MB27	child	Group 4	classic	NM	Negative	Negative	N/A
MB28	child	Group 4	classic	NM	Negative	Negative	N/A
MB31	infant	Group 4	classic	NM	Negative	Negative	N/A
MB33	child	Group 4	classic	NM	Negative	Negative	N/A
MB14	infant	Group 4	classic	NM	Negative	Negative	N/A
MB19	child	Group 4	classic	NM	Negative	Negative	N/A
MB38	child	Group 4	classic	NM	Negative	Negative	N/A
MB41	child	Group 4	classic	NM	Negative	Negative	N/A
MB42	child	Group 4	classic	NM	Negative	Negative	N/A
MB44	child	Group 4	classic	NM	Negative	Negative	N/A
MB18	child	Group 4	LCA	NM	Negative	Partially positive	Cytoplasm
MB10	child	N/A	classic	NM	Negative	Partially positive	Cytoplasm
MB4	infant	N/A	desmoplastic/nodular	NM	Negative	Partially positive	Cytoplasm

Adult: > 16 years, child: 4–16 years, infant: < 4 years.

ALCAM, activated leukocyte cell adhesion molecule; IHC, immunohistochemistry; M, male; F, female; N/A, data not available; MBEN, medulloblastoma with extensive nodularity; LCA, large cell/anaplastic; NM, no mutation.

^a^17–21 years.

^b^These cases were defined as WNT according to the mutation of *CTNNB1* and nuclear staining of β-catenin.

^c^Silent mutation.

**Fig 1:**
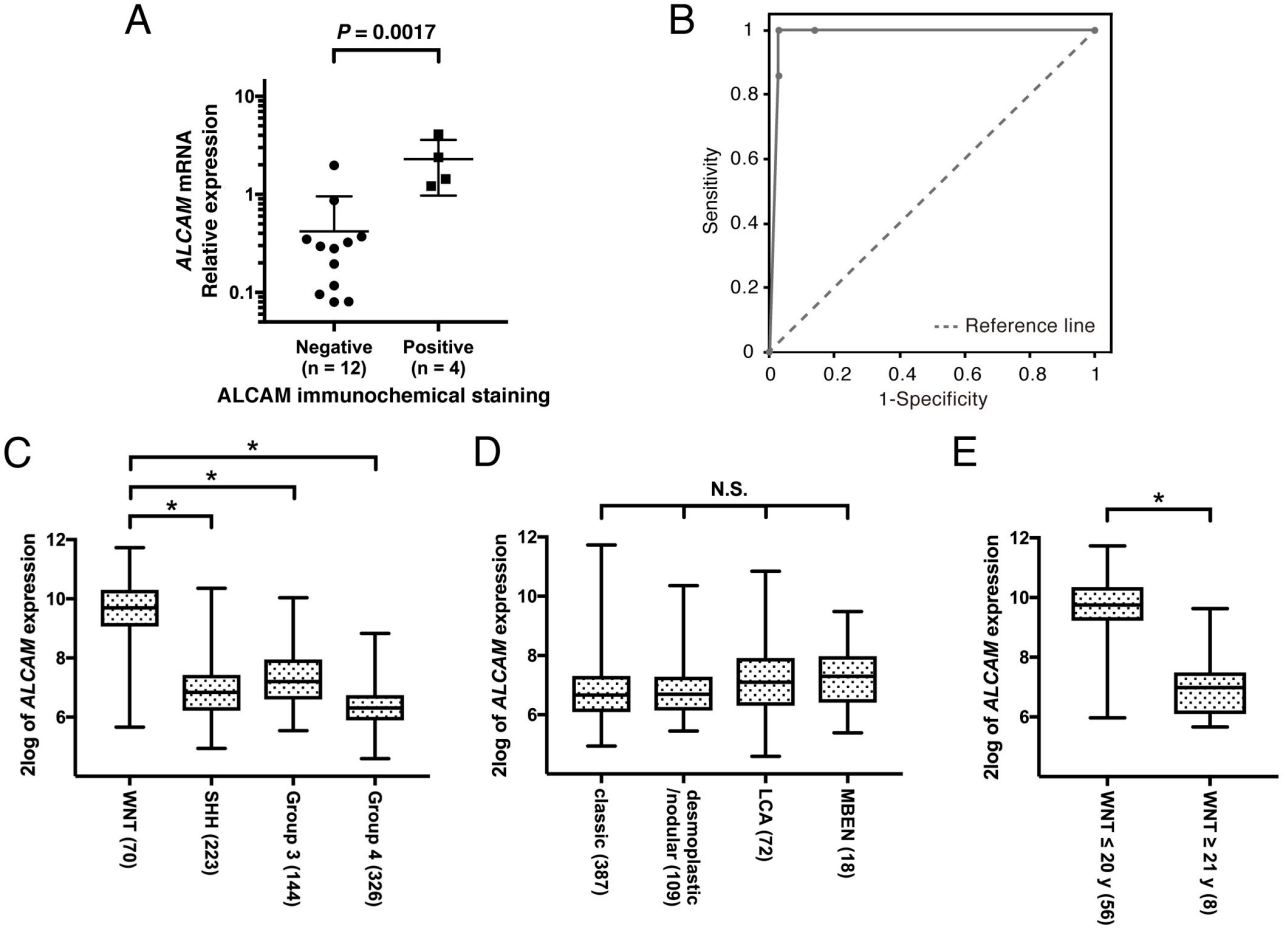
**(A)** Correlation between ALCAM immunohistochemical staining and *ALCAM* mRNA expression. ALCAM-positive medulloblastoma (MB) cases showed increased levels of *ALCAM* compared with the ALCAM-negative cases (*P* = 0.0017). The relative expression in human brain (cerebellum) was arbitrarily set at 1.0. **(B)** Receiver operating characteristic (ROC) curve for positive immunohistochemical expression of ALCAM in WNT subgroup. Area under the curve (AUC), 0.984. **(C–E)** *ALCAM* Expression in the Cavalli-763 MB cohort from the R2 Genomics Platform. **(C)** The WNT subgroup of MB strongly expressed *ALCAM* as compared with the other subgroups. **(D)** There was no clear correlation between *ALCAM* expression levels and histologic MB subtypes. **(E)** In the WNT subgroup of MB cases, ALCAM expression differed between the ages ≤ 20 y and ≥ 21 y. The box-and-whisker plots show the medians (thick horizontal lines) and interquartile ranges (IQRs; boundaries of the box) and ranges. *: *P* < 0.0001; N.S.: not significant.

To validate the correlation between the immunohistochemical staining data and *ALCAM* expression, we performed qPCR analysis of four ALCAM-positive and 13 ALCAM-negative cases. The mean relative expression level in the ALCAM-positive cases was 10 times higher than that in the ALCAM-negative cases (*P* = 0.0017; Fig 1A). These findings showed that *ALCAM* expression was higher in the WNT subgroup of MB than in the non-WNT subgroup. Furthermore, the correlation between ALCAM protein level determined by immunohistochemical staining and *ALCAM* mRNA expression was confirmed.

To evaluate the reliability of the WNT subgroup of MB using ALCAM immunohistochemical staining, ROC curves were prepared using the proportion of ALCAM-positive tumor cells (0, 1, 25, 50, 75, 100%), from representative tumor areas, as the independent variable (Fig 1B). Analyzing the seven cases in WNT subgroup and the 36 cases in the non-WNT subgroup, the AUC was 0.984, indicating a high accuracy, and the optimal cutoff was > 25% or > 50%.

Similar results were observed in the large cohorts of the Cavalli-763 MB dataset [24] from the R2: Genomics Analysis and Visualization Platform (http://r2.amc.nl). Specifically, *ALCAM* expression was significantly higher in the WNT subgroup of MB compared to that in the SHH, Group 3, and Group 4 subgroups (*P* < 0.0001; Fig 1C). On the other hand, no correlation was found between *ALCAM* expression and histologic subtypes of MB (Fig 1D). *ALCAM* expression was generally higher in the WNT subgroup, but this feature was not observed in cases of patients older than 21 y (mean, 33.2; range, 23–56 y; P < 0.0001; Fig 1E).

**Fig 2:**
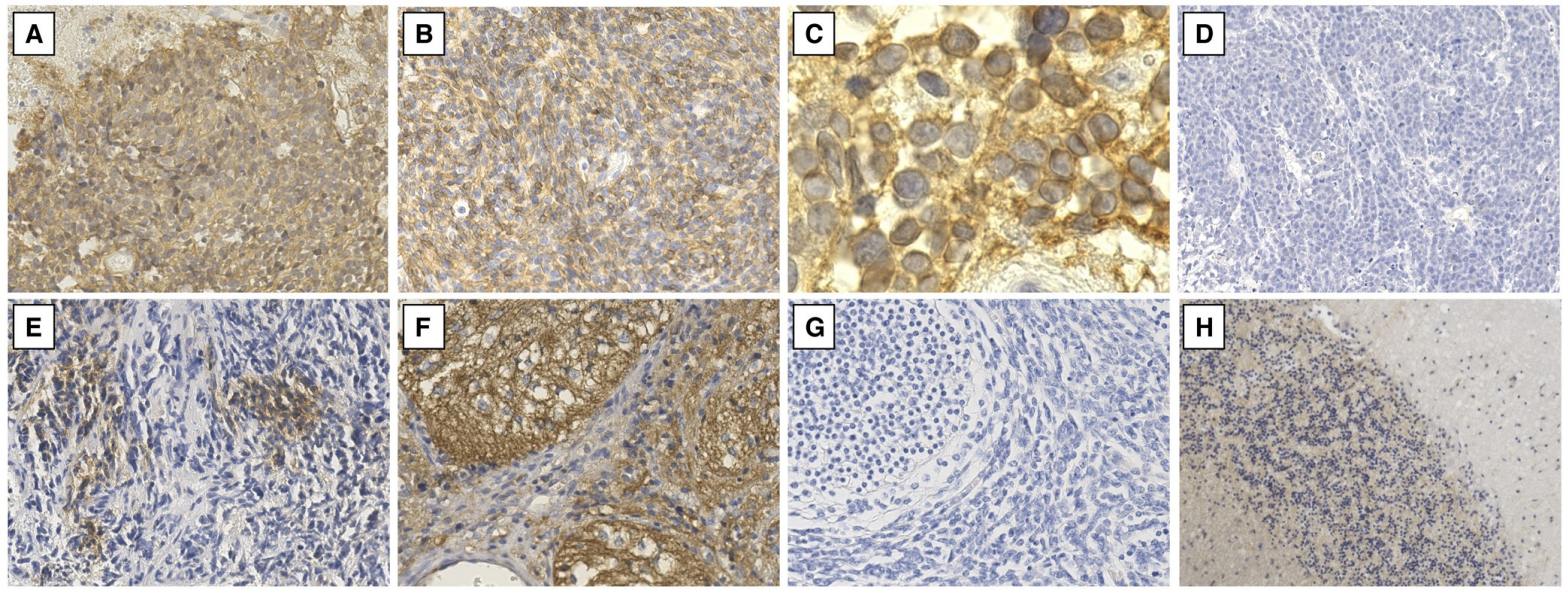
Immunohistochemical staining of ALCAM in medulloblastoma (MB) specimens. **(A, B)** ALCAM-positive cases: the WNT subgroup showed diffuse positive staining of ALCAM. **(C)** Most of the ALCAM-positive cells stained predominantly in the cytoplasm. **(D)** An ALCAM-negative case. **(E)** A partially positive case of ALCAM staining: few scattered ALCAM-positive cells can be observed. **(F)** The MBEN histological variant demonstrated a different ALCAM expression profile: positive staining in the lobular architectural region and negative in the internodular region. **(G)** The desmoplastic/nodular variant showed no ALCAM expression in most of the pale nodular areas and internodular areas. **(H)** In normal cerebellar tissue, the expression of ALCAM is not observed in the molecular layer, but is weakly detected in the granular layer and white matter. Original magnification, ×200 (A, B, D–G), ×1,000 (C) and ×100 (H).

In the seven WNT molecular subgroup cases, the tumor cells of the whole section were diffusely stained for ALCAM (Fig 2A and 2B). Most of the ALCAM-positive tumor cells stained predominantly in the cytoplasm, but a few of them showed some staining in the cell membrane (Fig 2C). In the majority of the SHH, Group 3, and Group 4 subgroup cases, no expression of ALCAM was observed in the tumor cells (Fig 2D). We also observed seven partially positive cases in which ALCAM-positive cells with cytoplasmic staining were observed in some areas of the tissue section, two Group 3 cases, three Group 4 cases, and two cases in the N/A group (Fig 2E). The MBEN histological subtype, observed in only one case, showed a characteristic staining pattern of neuropil-like tissue and differentiated neurocytic cells in the expanded lobular architecture stained positive for ALCAM (mainly in the membrane), while the small round neurocytic cells in the internodular areas were negative (Fig 2F). In contrast, in five desmoplastic/nodular cases, the tumor cells showing signs of variable neurocytic maturation in pale islands failed to show ALCAM staining, similar to the internodular areas (Fig 2G). However, in one desmoplastic/nodular case, weak ALCAM staining was observed in the tumor cells of a few pale islands. In the normal cerebellum, ALCAM staining was absent in the molecular layer, while the granular layer and white matter stained weakly (Fig 2H).

### Expression of ALCAM and its effect on cell proliferation and migration in MB cell lines

**Fig 3:**
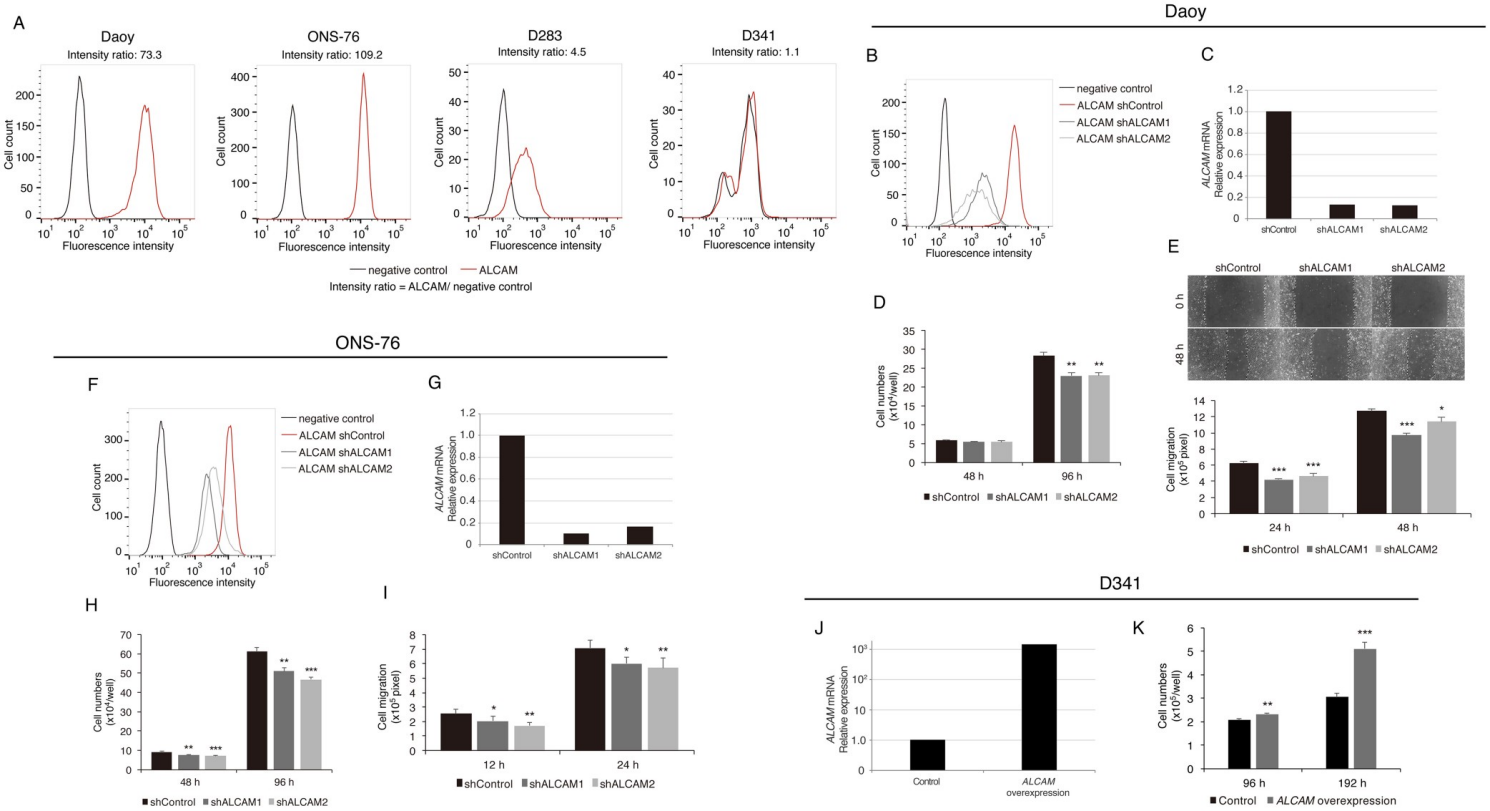
Expression of ALCAM in medulloblastoma (MB) cell lines and *in vitro* study of its function in Daoy and ONS-76 cells. **(A)** Four MB cell lines exhibited different ALCAM expression as measured by flow cytometry. **(B, C, F, G)** Knockdown of *ALCAM* in Daoy and ONS-76 cells was verified using flow cytometry (B, F) and qPCR (C, G). **(D, E, H, I)** *ALCAM* silencing inhibits the proliferation (D, H) and migration (E, I) of Daoy and ONS-76 cells. **(J)** Overexpression of *ALCAM* in D341 cells was confirmed by qPCR. **(K)** *ALCAM* overexpression promotes the proliferation of D341 cells. Data are reported as the mean ± SE. *: *P* < 0.05; **: *P* < 0.01; ***: *P* < 0.0001.

To evaluate the expression levels of ALCAM, we performed flow cytometry analysis of four human MB cell lines. ALCAM expression differed in each of the cell lines (Fig 3A) with the lowest levels being found in D341 cells (IR = 1.1) and the highest levels in Daoy and ONS-76 cells (IR = 73.3 and 109.2, respectively). Based on these results, we selected Daoy cells as a representative cell line with high ALCAM expression to investigate the functional role of ALCAM in MB. Two ALCAM-depleted Daoy cell lines were generated by RNA interference and the reduction of ALCAM expression was confirmed by flow cytometry analysis and qPCR (Fig 3B and 3C, respectively). The comparison between ALCAM-depleted and control Daoy cells showed that ALCAM depletion decreased cell proliferation and migration (Fig 3D and 3E, respectively). Specifically, there was no difference in cell proliferation at 48 h, while a difference was observed at 96 h, with the mean numbers (± SE) of the shControl, shALCAM1, and shALCAM2 Daoy cells being 28.3 ± 0.879, 22.9 ± 0.970, and 23.1 ± 0.798 × 10^4^ cells, respectively (Fig 3D). Additionally, in wound healing assays, shALCAM1 and shALCAM2 Daoy cells showed 32% and 26% reduced migration at 24 h, respectively, and 24% and 11% at 48 h, respectively, compared to that of the shControl Daoy cells (Fig 3E). Similar results were observed with the ALCAM-depleted ONS-76 cell lines (Fig 3F–3I). These results demonstrated that ALCAM acted as a positive regulator of proliferation and migration in Daoy and ONS-76 cells *in vitro*.

Furthermore, D341 cells, which had the lowest levels of ALCAM expression, were used to establish an *ALCAM*-overexpressing D341 cell line using cDNA transduction. Increased *ALCAM* expression was confirmed by qPCR analysis (Fig 3J). Comparison of *ALCAM*-overexpressing D341 cells and control D341 cells revealed that enhanced *ALCAM* expression resulted in increased cell proliferation (Fig 3K).

### Depletion of ALCAM affects invasion at dissemination sites in an orthotopic mouse model of MB

**Fig 4:**
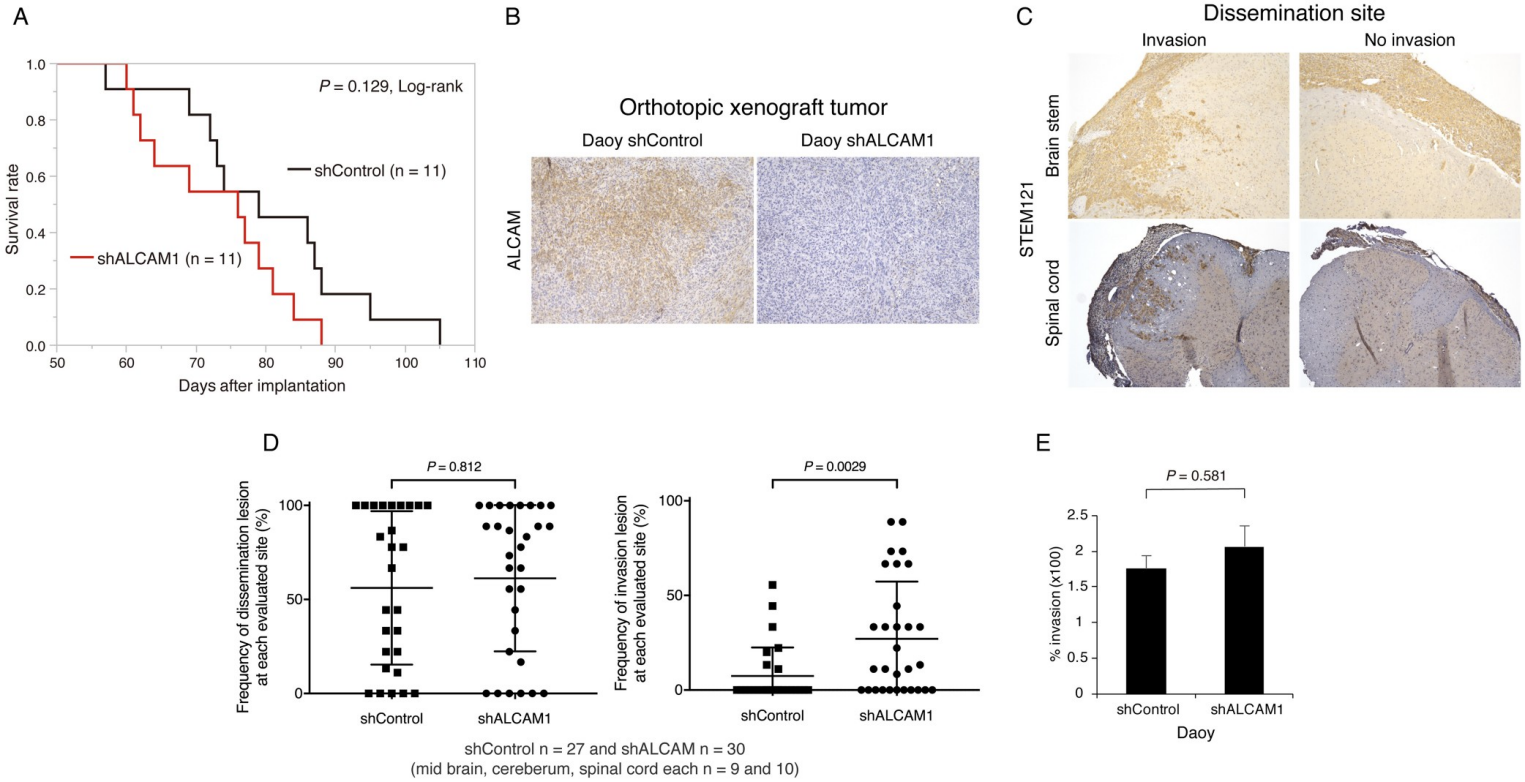
Functional analysis of ALCAM in an orthotopic mouse model and *in vitro* transwell assay in Daoy cells. **(A)** Kaplan–Meier survival curves of mice injected with *ALCAM*-silenced and control Daoy cells showed no significant difference between the two groups (*P* = 0.129, log-rank test). **(B)** Representative immunohistochemical staining of ALCAM in primary tumors in the cerebellum. **(C)** Representative STEM121 staining showing invasive and noninvasive tumor cells in disseminated lesions. **(D)** The frequency of dissemination in the *ALCAM*-silenced and control group was similar (*P* = 0.812). The frequency of invasion observed at the dissemination sites was significantly higher in the *ALCAM*-silenced group than that in the control group (*P* = 0.0029). **(E)** The invasion percentage of ALCAM-silenced and control Daoy cells was not significantly different, as per the results of the transwell assay (*P* = 0.581). Original magnification, ×100 (B, C).

Next, we assessed the effects of ALCAM on tumor progression and dissemination *in vivo*. Stably transfected ALCAM-depleted or control Daoy cells were inoculated into the right cerebellar hemisphere of mice. Tumors developed in both groups of mice. During the experiment, 9 mice in the shControl were euthanized and two were found dead while 10 mice in the shALCAM group were euthanized and one mouse was found dead. Although there was no significant difference in the survival rates between the shControl and shALCAM groups (*P* = 0.129, log-rank test), the survival of mice injected with ALCAM-depleted cells appeared to be shorter than that of the control mice (Fig 4A). Upon pathological evaluation of the brains and spinal cords of the mice, the primary tumors were found in the cerebellum and leptomeningeal disseminated lesions were found on the surface of the cerebrum, brain stem, and spinal cord. Immunostaining of ALCAM confirmed that depletion of ALCAM was maintained in the shALCAM group (Fig 4B). In the disseminated lesions, several foci of invasive tumor cells were found into the subpial parenchyma (Fig 4C). The frequency of sections with dissemination lesions in the two groups was similar (*P* = 0.812; Fig 4D). However, invasion at the dissemination sites was observed more frequently in the shALCAM group compared to that in the shControl group (*P* = 0.0029; Fig 4D). These results indicated that ALCAM depletion may be associated with a more invasive tumor-cell phenotype *in vivo*. On the other hand, there was no significant difference in the invasiveness of shControl and shALCAM1 Daoy cells as per the results of the *in vitro* Transwell assay. The invasion percentage (± SE) of the shControl and shALCAM1 Daoy cells was 176 ± 18.5 and 206 ± 29.8, respectively (*P* = 0.581; Fig 4E).

## Discussion

In the current study, we first showed that ALCAM expression was strongly correlated to the WNT molecular subgroup of MB. Few studies have investigated the expression of ALCAM in MB; among them, one study assessed ALCAM levels in various primary central nervous system tumors, including MB [20]. In that study, Allmendinger et al [20] showed that the positive rate of ALCAM in MB is 31%, similar to that obtained in our study. However, the authors did not examine the relation between ALCAM expression and the molecular subgroup or histological subtypes [20].

Recent studies have focused on the molecular classification of MB and the expression of several genes related to molecular subgroups has been determined in MB. However, there is no report investigating the relationship between ALCAM and the WNT subgroup. Immunohistochemically, the nuclear staining of β-catenin resulting from a *CTNNB1* mutation is associated with the WNT subgroup and is a good diagnostic tool for clinical applications [25]. However, immunohistological diagnosis of the WNT subgroup of MB using β-catenin nuclear staining alone is considered insufficient [26]. ALCAM has the potential of being a new WNT-related biomarker for improving the reliability of a WNT subgroup diagnosis of MB.

Interestingly, it has been recently reported that there is a strong correlation between nuclear staining of β-catenin and ALCAM levels in adamantinomatous craniopharyngioma [27], which is closely related to the Wnt signal pathway [28]. This report and our current results suggest a relationship between the Wnt signal pathway and ALCAM expression. It is known that embryonic expression of ALCAM occurs through non-canonical Wnt/JNK signaling [29, 30]. However, it is unclear whether the activation of the Wnt pathway caused by the *CTNNB1* mutation is associated with ALCAM expression.

Despite the fact that ALCAM was originally identified as a membrane protein, the localization of ALCAM staining in MB was mainly cytoplasmic and only partially observed in the membrane. This is consistent with a previous report by Allmendinger et al [20]. In fact, ALCAM is known to be expressed in the cytoplasm of many carcinomas and the pattern of expression seems to vary depending on the particular carcinoma [11, 13, 19, 20]. There is also a report that cell function is altered by the translocation of ALCAM from the membrane to the cytoplasm [13].

Regarding the immunohistochemical evaluation of ALCAM, it is necessary to understand ALCAM expression in the normal brain. As we have shown, ALCAM was slightly expressed in the granular layer of the cerebellum. Furthermore, Allmendinger et al [20] have reported that ALCAM is expressed in the normal cerebral tissue, such as hippocampus and basal ganglia, and also in reactive glial cells. In the current study, we performed Immunohistochemical evaluation of ALCAM in representative tumor areas.

In our functional study using MB cell lines, we demonstrated that *in vitro* silencing of ALCAM in Daoy and ONS-76 cells inhibited cell proliferation and migration. Meanwhile, the overexpression of ALCAM in D341 cells promoted cell proliferation, although cell migration could not be evaluated due to the fact that the cells grow in suspension and are not adherent cells. Our findings indicate that ALCAM may act as a positive regulator of cell proliferation in MB. However, we obtained unexpected results in the orthotopic mouse model, where the survival of mice injected with ALCAM-silenced Daoy cells appeared to be shorter than that of the control mice, even though the difference between the two groups was not statistically significant. We also found that ALCAM depletion in Daoy cells increased invasion at disseminated sites in the orthotopic mouse model. On the other hand, the increased invasiveness of ALCAM-depleted Daoy cells was not observed in the context of an *in vitro* transwell assay. These results seem contradictory, but the difference between ALCAM function *in vitro* and *in vivo* indicates that ALCAM may be affected by the surrounding microenvironment. Notably, our results are consistent with those from previous reports regarding other carcinomas in which depletion of ALCAM suppresses cell proliferation and migration *in vitro*, but the cells demonstrate invasiveness *in vivo* [14, 16, 19, 31]. For example, in cell lines of malignant mesothelioma and endometrioid endometrial cancer, proliferation and migration are inhibited *in vitro* by ALCAM silencing [16, 19]. Nevertheless, ALCAM-negativity at the invasive front of the tumor has been reported as a marker of myometrial invasion in tissue of endometrioid endometrial cancer [31]. Moreover, in a metastatic melanoma cell line, interfering with endogenous ALCAM through the expression of an amino-terminally truncated ALCAM, which disrupts ALCAM–ALCAM interactions, increases cell invasive growth in skin reconstructions [14]. The attenuation of ALCAM enhances the invasive abilities of the tumor, which is arguably caused by the reduction of ALCAM-mediated adhesion, resulting in less adhesive and more mobile tumor cells [14, 31]. Therefore, the functional role of ALCAM might vary depending on the surrounding microenvironment.

ALCAM has been shown to be a prognostic marker in several types of cancers; however, it is both a marker of good and poor prognosis depending on the cancer type [9–13]. In our study, the expression of ALCAM was observed particularly concentrated in the WNT subgroup of MB, which typically shows a good long-term prognosis in comparison to the other subgroups. Therefore, ALCAM may be regarded as a good prognostic marker in MB. Conversely, a lack of ALCAM may contribute to poor prognosis. Differences in ALCAM expression levels might be related to a factor regulating the cell kinetics between WNT MB and non-WNT MB. Accordingly, we showed that ALCAM silencing enhanced the invasiveness of disseminated lesions in the orthotopic mouse model. The invasion ability at disseminated lesions is an important negative prognostic factor of MB as most MB recurrence is caused by leptomeningeal dissemination.

A limitation of our current study was the relatively small sample size of our cohort compared to that of other large cohorts. The small sample size used for ALCAM immunohistochemical staining may have resulted in inevitable statistical bias. However, there was no significant difference in our molecular subgrouping results compared to that using another cohort [4]. It is noteworthy that our study lacked a validation study of the ALCAM immunohistochemical staining, and thus our immunohistochemical findings may be insufficient to assert ALCAM as a biomarker for the WNT subgroup of MB. However, we did perform a comparative analysis using a large cohort (Cavalli-763 MB dataset) [24] of the R2: Genomics Analysis and Visualization Platform. This comparative analysis was based on gene expression and showed a strong correlation between *ALCAM* expression and the WNT subgroup of MB. Consistent with this, our study showed a correlation between ALCAM protein levels based on immunohistochemical staining and *ALCAM* mRNA gene expression levels analyzed by RT-qPCR. As evaluation of patient age and pathological type was insufficient in our cohort, we examined this using expression data from a large cohort of the R2 database. Analysis of the R2 database cases revealed *ALCAM* expression was low in patients 21 y and older, even in the WNT subgroup with high *ALCAM* expression. Therefore, we believe that limiting the evaluation of cases to patients 20 years or younger improves the strength of the correlation between ALCAM and the WNT subgroup.

According to the results from the analysis of clinical sample in which ALCAM was strongly expressed in the WNT subgroup, it may be more appropriate to use a WNT subgroup cell line. However, cell lines belonging to the WNT subgroup are not readily available [32]. Daoy and ONS-76 cells used in our study strongly express ALCAM and have been considered to be of the SHH subgroup, while D341 and D231 cells weakly express ALCAM and are considered to be of the Group 3 and Group 3/4 subgroups, respectively [32]. In our current study, positive ALCAM immunostaining was observed in only one of the eight cases of the SHH subgroup and *ALCAM* expression was low. Further studies are required to determine whether ALCAM is strongly expressed in the SHH subgroup cell line, despite low expression of ALCAM in the SHH subgroup clinical sample.

## Conclusions

Clinical sample analysis performed in our current study suggested that ALCAM expression in MB was strongly correlated to the molecular subgroup. Specifically, ALCAM was highly expressed in the WNT subgroup of MB compared to that in non-WNT subgroups of MB. Furthermore, the cell kinetics of MB cell lines were altered by ALCAM expression. ALCAM appeared to be involved as a positive regulator of proliferation and migration of MB tumor cells *in vitro*. However, *in vivo* studies using an orthotopic mouse model demonstrated that the attenuation of ALCAM enhanced cell invasiveness in disseminated lesions.
