# Supplementary material for: Activated leukocyte cell adhesion molecule expression correlates with the WNT subgroup in medulloblastoma and is involved in regulating tumor cell proliferation and invasion
Source: PLoS One. 2020 Dec 3;15(12):e0243272. doi: 10.1371/journal.pone.0243272 (PMC7714159; doi:10.1371/journal.pone.0243272)
Supplement: Attachment — Submitted filename: ReviewCommentsAuthors_PONE-D-19-17600.pdf [file pone.0243272.s001.pdf]

The expression of activated leukocyte cell adhesion molecule correlates with the WNT subgroup in medulloblastoma and is involved in the regulation of tumor cell invasion

Takamune Achiha; Noriyuki Kijima; Yoshinori Kodama; Naoki Kagawa; Manabu Kinoshita; Yasunori Fujimoto; Masahiro Nonaka; Namiko Nishida; Takumi Yamanaka; Atsuko Harada; Kanji Mori; Junya Fukai; Naohiro Tsuyuguchi; Takehiro Uda; Kenichi Ishibashi; Yusuke Tomogane; Daisuke Sakamoto; Tomoko Shofuda; Ema Yoshioka; Daisuke Kanematsu; Masayuki Mano; Betty Luu; Michael D. Taylor; Yonehiro Kanemura; Haruhiko Kishima

The aim of the present study was to reveal the functional role and significance of ALCAM expression in Medulloblastoma (MB). In the first part of the results, the authors carried out several functional *in vitro* and *in vivo* assays by silencing ALCAM using RNA interference in order to unveil the function of ALCAM in MB. In the second part of the study, the authors provide new information about ALCAM expression correlation with MB molecular and histological subtypes in a MB FFPE cohort conformed by 39 patients. As a conclusion, authors propose ALCAM as a novel MB WNT biomarker.

Reviewers Concerns:

- Authors should declare that the clinical stratification of MB tumors is stated at the 2016 Consensus Paper (Ramaswamy, V *et al.* Acta Neuropathol (2016) 131:821–831).
- The article seems to report two well-differentiated studies aimed at: i) the functional role of ALCAM in MB models and ii) the potential use of ALCAM expression as an IHC biomarker in MB, however, both studies are incomplete. Additional analyses are necessary to clarify the role of ALCAM as well as to propose ALCAM as a potential biomarker for the MB - WNT subgroup.
- Review of the Functional Analyses:
    - Author should explain why MB cell lines were cultured in different medium and FBSi conditions. This could be a source of variability.
    - Results reported in Fig1H show differences of proliferation that are hardly appreciable for the ONS-76 cell lines at 48h. Author do not specify the significance of the *** symbol.
    - Authors should perform additional assays using different MB cell lines, in order to explore further the effect of ALCAM depletion on migration/invasion, and clarify the contradictory findings observed between in vitro and in vivo assays.
- Review of the IHC analysis:
    - The contents of the ALCAM expression analysis section should be placed before the functional part, making it easier to understand the rational of the study and to follow the results.
    - The MB FFPE cohort conformed by 36 cases used in the study is far too small to support the conclusions and includes both pediatric and adult MB cases. Only six of these are WNT MBs. The authors should prove their findings in a larger cohort.
    - Table 1, ALCAM positive staining was observed in two cases (one SHH) with negative nuclear beta-catenin expression. This is critical for the study and questions the ALCAM staining as a reliable marker. Authors must verify the presence of CTNNB1 mutation.
    - In addition, no biomarker studies are acceptable if the authors do not validate their results in an independent cohort.
    - MB is rarely diagnosed in adults, whereas MB is the most common malignant pediatric brain tumor. However, four of the six MB WNT cases included in the FFPE cohort were

adults, only two children. Adult MB is distinct from childhood MB with clear differences in the molecular variants and clinical evolution, and should thus be analysed separately. Authors do not address this issue. The cohort is biased and does not fully represent the MB WNT subgroup.

- Authors describe the IHC pattern of ALCAM in WNT-MB samples predominantly in the cytoplasm, but also the presence of some staining in the cell membrane. If authors propose the use of ALCAM as a MB-WNT biomarker, the IHC staining pattern must be described accurately (see Mezzanzanica, D et al. Clin Cancer Res 2008;14(6)). IHC consistent controls should be reported.
- Studies that propose possible biomarkers should include analyses such as ROC curves to demonstrate the reliability and validity of the results.
- The correlation between ALCAM IHC staining and ALCAM mRNA expression by qPCR was performed in a limited number of samples: 3 positive vs 6 negative for ALCAM IHC.
- The ALCAM mRNA validation in Cavalli microarray cohort (n=763) showed a large variability of ALCAM expression within subgroups and overlap across MB subgroups. ALCAM mRNA expression in MB subgroups has low specificity based on the presented results.
- Authors did not assess the correlation between ALCAM expression and the MB histological subtypes as the cohort does not has a fully representation of all histological MB subtypes. Authors should enlarge the cohort all MB histological subtypes in order to obtain robust conclusions.
- The IHC cut-off values for subdivision must be more restrictive in a biomarker study to be reliable.

- The results are insufficient to support the exposed conclusions.
- There are several typos and grammatical errors. For example: “Table1: Adult (≥ 16 yeas)” instead of years; The correct spelling is “partially” not “partial” in the staining pattern description; In page 5 line 88 “many names” is used instead of “alias”; “Subdivision” is used instead of “subgroups”).
