# Supplementary material for: Activated leukocyte cell adhesion molecule expression correlates with the WNT subgroup in medulloblastoma and is involved in regulating tumor cell proliferation and invasion
Source: PLoS One. 2020 Dec 3;15(12):e0243272. doi: 10.1371/journal.pone.0243272 (PMC7714159; doi:10.1371/journal.pone.0243272)
Supplement: Attachment — Submitted filename: ReviewerAttachments.pdf [file pone.0243272.s003.pdf]

R1_D-19-17600

Activated leukocyte cell adhesion molecule expression correlates with the WNT subgroup in medulloblastoma and is involved in regulating tumor cell proliferation and invasion

Takamune Achiha, Noriyuki Kijima, Yoshinori Kodama, Naoki Kagawa, Manabu Kinoshita, Yasunori Fujimoto, Masahito Nonaka, Namiko Nishida, Takumi Yamanaka, Atsuko Harada, Kanji Mori, Junya Fukai, Naohiro Tsuyuguchi, Takehiro Uda, Kenichi Ishibashi, Yusuke Tomogane, Daisuke Sakamoto, Tomoko Shofuda, Ema Yoshioka, Daisuke Kanematsu, Masayuki Mano, Betty Luu, Michael D. Taylor, Yonehiro Kanemura, Haruhiko Kishima.

The aim of the present study was to reveal the functional role and significance of ALCAM expression in Medulloblastoma (MB). The authors provide new information of ALCAM protein levels analysed by IHC in a MB FFPE cohort conformed by 36 patient and performed an *in silico* correlation between ALCAM gene expression and the MB molecular and histological subtypes as well as patient age's in a large MB cohort from the R2 genomics Platform (Cavalli *et al.* Cancer Cell 2017). In the second part of the study, the authors carried out several functional *in vitro* and *in vivo* assays by silencing ALCAM using RNA interference in order to unveil the function of ALCAM in MB. As a conclusion, authors offer ALCAM as a novel MB WNT biomarker.

Reviewer Concerns:

There has been a qualitative improvement in the manuscript compared to the previous version. The authors have restructured the article and now the results are easier to follow, however, the results are still insufficient to support the conclusions presented. The article is attractive and unveils an interesting role of ALCAM in MB-WNT subgroup, but it has to be reported as a study of the MB underlying biology, not as a biomarker study.

Major Comments:

- The MB FFPE cohort conformed by 36 cases used in the study is far too small to support the conclusions. The authors should prove their findings in a larger cohort. The strength of the preliminary data (IHC of 36 samples) is not sufficient to support the rationale of ALCAM as a WNT-related biomarker.
- Biomarker studies are not acceptable if authors do not validate their results in an independent cohort using the same technique. In this case, the independent validation the authors provide is an *in silico* validation (Cavalli *et al.* Cancer Cell 2017) at the gene expression level. Authors do not validate their results at the protein level using the IHC technique or other method to detect protein levels. The *in silico* analysis suggests a tendency but not a validation itself.
- The authors propose ALCAM IHC as an additional WNT-related biomarker to β-catenin to improve the reliability of diagnosis. However, ALCAM IHC is positive in six WNT-MB (n=6/6, 100% of all WNT analyzed) and presents an inconsistent positive result in one SHH (n=1/5, 20% of all SHH analyzed). This is not acceptable in a small cohort of 36 samples if the authors pretend to propose ALCAM as a biomarker.
- The IHC training cohort of 36 FFPE patients is small and not fully characterized:
    a. 2 NA Molecular subgroup
    b. 5 NA CCNB1 status

Minor Comments

- There is much more updated MB literature than the referenced: Northcott, P.A., Robinson, G.W., Kratz, C.P. *et al.* Medulloblastoma. *Nat Rev Dis Primers* **5,** 11 (2019). https://doi.org/10.1038/s41572-019-0063-6
- Authors declare that ALCAM depletion is associated with a more invasive tumour cells phenotype *in vivo*. This could be confirmed by a transwell migration assay analysis.
- Figure 2H: ALCAM was “weakly” detected in normal cerebellum in granular layer and white matter by IHC. The partially IHC staining of ALCAM in normal tissues should be further explored in normal cerebellum as well as brain if authors propose ALCAM as a possible WNT-related biomarker.
- Positive ALCAM IHC staining of one of the five SHH tumor samples included in the study as well as high ALCAM mRNA levels in SHH cell lines (DAOY and ONS-76) should be declared and further investigated in order to understand the role of ALCAM in MB.
