# Supplementary material for: Activated leukocyte cell adhesion molecule expression correlates with the WNT subgroup in medulloblastoma and is involved in regulating tumor cell proliferation and invasion
Source: PLoS One. 2020 Dec 3;15(12):e0243272. doi: 10.1371/journal.pone.0243272 (PMC7714159; doi:10.1371/journal.pone.0243272)
Supplement: Attachment — Submitted filename: R2_D-19-17600_20201106.pdf [file pone.0243272.s005.pdf]

R2_D-19-17600

The expression of activated leukocyte cell adhesion molecule correlates with the WNT subgroup in medulloblastoma and is involved in the regulation of tumor cell invasion

Takamune Achiha; Noriyuki Kijima; Yoshinori Kodama; Naoki Kagawa; Manabu Kinoshita; Yasunori Fujimoto; Masahiro Nonaka; Namiko Nishida; Takumi Yamanaka; Atsuko Harada; Kanji Mori; Junya Fukai; Naohiro Tsuyuguchi; Takehiro Uda; Kenichi Ishibashi; Yusuke Tomogane; Daisuke Sakamoto; Tomoko Shofuda; Ema Yoshioka; Daisuke Kanematsu; Masayuki Mano; Betty Luu; Michael D. Taylor; Yonehiro Kanemura; Haruhiko Kishima

The aim of the present study was to reveal the functional role and significance of ALCAM expression in Medulloblastoma (MB). The authors provide new information of ALCAM protein levels analysed by IHC in a MB FFPE cohort conformed by 45 patient and performed an *in silico* correlation between ALCAM gene expression and the MB molecular and histological subtypes as well as patient age's in a large MB cohort from the R2 genomics Platform (Cavalli *et al.* Cancer Cell 2017). In the second part of the study, the authors carried out several functional *in vitro* and *in vivo* assays by silencing ALCAM using RNA interference in order to unveil the function of ALCAM in MB.

Reviewer Concerns:

There has been an improvement in the manuscript compared to the previous version. The authors have ensured that they did not define ALCAM as a WNT-related biomarker and the Results are now presented as an interesting correlation between ALCAM expression and the MB-WNT subgroup. The article is not as a biomarker study but an attractive exploration of the MB underlying biology and unveils an interesting role of ALCAM in MB-WNT subgroup. However, some points must be clarified.

Minor Comments:

- Authors need to clarify the finality and the description of the ROC curve analysis. It is not clear if the ROC curve analysis is meant to select ALCAM IHC positivity cut-point neither how many MB samples are used or their subgroup. Authors must remove the word "diagnosis" from the manuscript in line 325 "To evaluate the reliability of the WNT subgroup diagnosis of MB using ALCAM" as it is misleading.
