# Supplementary material for: Activated leukocyte cell adhesion molecule expression correlates with the WNT subgroup in medulloblastoma and is involved in regulating tumor cell proliferation and invasion
Source: PLoS One. 2020 Dec 3;15(12):e0243272. doi: 10.1371/journal.pone.0243272 (PMC7714159; doi:10.1371/journal.pone.0243272)
Supplement: Attachment — Submitted filename: Response_to_Reviewers.docx [file pone.0243272.s002.docx]

Editor

(1) ALCAM overexpression experiments

Response: Related to #1-1, we have performed ALCAM overexpression experiments using the D341 cell line and showed that ALCAM promotes cell proliferation (Fig 3J and 3K).

(2) Presentation of ROC curve and description of cut-off value for ALCAM immunohistochemistry

Response: Related to #2-10, we have created ROC curves and evaluated the diagnostic performances of ALCAM Immunohistochemical staining and have identified optimal cut-off points for the diagnosis of the WNT molecular subgroup (Fig 1B).

(3) Discussion related to ALCAM immunohistochemistry, such as (i) ALCAM immunostaining pattern, (ii) ALCAM-positive and nuclear ß-catenin-negative cases, (iii) ALCAM mRNA expression levels in medulloblastoma subgroups

Response: Related to #2-9, #2-11, and #2-12, we described the immunostaining patterns in all ALCAM-positive staining cases in Table 2. Moreover, we examined the histologic subtype and age group of MB with respect to the expression of ALCAM (Fig 1D and 1E).

(4) Discussion of weak points in this report, such as (i) small sample size and (ii) lack of validation study. In addition, the authors need to discuss statistical power of a clinical study based on 36 cases of medulloblastoma patients.

Response: Related to #2-6, we have described these as limitations in the Discussion section.

(5) Language edition

Response: Related to #2-13, we have proofread the entire manuscript.

(6) Other issues pointed out by Reviewers

Response: We have responded to all reviewer questions as described below.

Reviewer #1

#1-1: They didn’t evaluated the effect of an overexpression induced in cell lines (mainly in the cell lines exhibiting a low expression level of ALCAM, D341, and D283) which should show an increasing rate of cell proliferation and migration (D341, and D28 proliferation and migration rate in normal conditions are not described).

Response: The ALCAM overexpression experiments using the D341 cell line showed that enhanced ALCAM expression increased cell proliferation. D341 cells migration could not be evaluated due to the feature that D341 cells grow in suspension and not as adherent monolayers. This has been noted in the revised manuscript.

#1-2: It is well known that Daoy cell line exhibits mutated p53 which is tightly related to beta catenin pathway. The Authors could analyze the p53 mutation status also in Daoy induced MB (p53 wild type or not) to give a hypothesis to explain the different results among different experimental conditions (“in vitro” and ”in vivo”)

Response: We analyzed the TP53 gene mutation status both *in vitro* in Daoy cell lines (shControl, shALCAM1, and shALCAM2) and in vivo in Daoy tumor tissues (shControl and shALCAM1). Sanger sequencing analysis of the Exon 2-11 region detected a common c.725G>T: p.C242F homozygous mutation in the *TP53* gene of all samples. This was consistent with the known mutation reported in Daoy line (Saylors et al., 1991).

Reviewer #2

#2-1: Authors should declare that the clinical stratification of MB tumors is stated at the 2016 Consensus Paper (Ramaswamy, V et al. Acta Neuropathol (2016)

131V821–831).

Response: As suggested, we have added this to the Introduction of the revised manuscript and provided the proper in-text citation of this consensus paper.

#2-2: Author should explain why MB cell lines were cultured in different medium and FBSi conditions. This could be a source of variability.

Response: We used the media and FBS conditions recommended for each cell line. Culturing may not be successful for a particular cell line when conditions are changed.

#2-3: Results reported in Fig1H show differences of proliferation that are hardly appreciable for the ONS-76 cell lines at 48h. Author do not specify the significance of the *** symbol.

Response: We apologize for the error in defining the sign of the significance. We revised the description from "*: P <0.05; **: P <0.0001" to "*: P < 0.05; **: P < 0.01; ***: P < 0.0001" and revised the figure as well.

#2-4: Authors should perform additional assays using different MB cell lines, in order to explore further the effect of ALCAM depletion on migration/invasion, and clarify the contradictory findings observed between in vitro and in vivo assays.

Response: We performed an ALCAM overexpression experiment using the D341 cell line. Enhanced ALCAM expression increased cell proliferation. An orthotopic mouse model using ONS-76 cells was also evaluated, but meningeal dissemination was not well formed.

#2-5: The contents of the ALCAM expression analysis section should be placed before the functional part, making it easier to understand the rational of the study and to follow the results.

Response: As the reviewer suggested, we have restructured the appropriate sections in the revised manuscript.

#2-6: The MB FFPE cohort conformed by 36 cases used in the study is far too small to support the conclusions and includes both pediatric and adult MB cases. Only six of these are WNT MBs. The authors should prove their findings in a larger cohort.

&

In addition, no biomarker studies are acceptable if the authors do not validate their results in an independent cohort.

Response: Due to sample limitations, our cohort size was relatively small and our study lacked a validation component using ALCAM immunohistochemical staining. However, analysis from a large cohort (the Cavalli-763 MB dataset of the R2: Genomics Analysis and Visualization Platform) based on gene expression analysis showed a strong correlation between ALCAM expression and the WNT subgroup of MB. We have included the small sample size our cohort as a limitation of our study in the Discussion of the revised manuscript.

#2-7: Table 1, ALCAM positive staining was observed in two cases (one SHH) with negative nuclear beta-catenin expression. This is critical for the study and questions the ALCAM staining as a reliable marker. Authors must verify the presence of CTNNB1 mutation.

Response: As shown in Table 1, we have verified CTNNB1 mutations in most of the cases used in our study.

#2-8: MB is rarely diagnosed in adults, whereas MB is the most common malignant pediatric brain tumor. However, four of the six MB WNT cases included in the FFPE cohort were adults, only two children. Adult MB is distinct from childhood MB with clear differences in the molecular variants and clinical evolution, and should thus be analysed separately. Authors do not address this issue. The cohort is biased and does not fully represent the MB WNT subgroup.

Response: MB is pediatric brain tumor, but the age distribution differs for each molecular subgroup. Adults (> 16 y) are not uncommon in WNT, SHH, and Group 4 subgroups of MB. In the WNT subgroup, infant cases (< 4 y) are rare and cases of children (4-16 y) and adults (>16 years) occur at a 2:1 ratio (Kool et al., 2012 and Taylor et al., 2012). In our cohort, the cases of the WNT subgroup included two children (6 and 7 years old) and four adults (16, 17, 18, and 20 years old) according to the original age distribution definition (Adult: ≥ 16 years, child: < 16 and ≥ 3 years, and infant: < 3 years). We have changed the definition of age distribution in the revised manuscript to be consistent with other cohorts (Adult: > 16 years, child: 4-16 years, infant: < 4 years). The cases of the WNT subgroup now included three children (6, 7, and 16 years old) and three adults (17, 18, and 20 years old). There was no significant difference in the age distribution of the other cohorts and both the child and adult cases were positive for ALCAM immunohistochemical staining.

#2-9: Authors describe the IHC pattern of ALCAM in WNT-MB samples predominantly in the cytoplasm, but also the presence of some staining in the cell membrane. If authors propose the use of ALCAM as a MB-WNT biomarker, the IHC staining pattern must be described accurately (see Mezzanzanica, D et al. Clin Cancer Res 2008;14(6)). IHC consistent controls should be reported.

Response: As suggested, we have added the information regarding ALCAM IHC pattern to Table 2 in the revised manuscript.

#2-10: Studies that propose possible biomarkers should include analyses such as ROC curves to demonstrate the reliability and validity of the results.

&

The IHC cut-off values for subdivision must be more restrictive in a biomarker study to be reliable.

Response: We have included the accuracy of the WNT subgroup diagnosis by ALCAM immunohistochemical staining and the optimal cutoff value according to ROC curve analysis.

#2-11: The ALCAM mRNA validation in Cavalli microarray cohort (n=763) showed a large variability of ALCAM expression within subgroups and overlap across MB subgroups. ALCAM mRNA expression in MB subgroups has low specificity based on the presented results.

Response: As pointed out, in a large cohort used for validation (the Cavalli-763 MB dataset), ALCAM expression in some cases exhibited overlap between MB subgroups. As a result of additional analysis, it became clear that the cases where ALCAM expression was low in the WNT subgroup, the patients were more than 21 years old. We have described this in the Results of the revised manuscript and added it as a limitation of our study.

#2-12: Authors did not assess the correlation between ALCAM expression and the MB histological subtypes as the cohort does not has a fully representation of all histological MB subtypes. Authors should enlarge the cohort all MB histological subtypes in order to obtain robust conclusions.

Response: In our small cohort, the histologic subtype was biased and difficult to evaluate. However, there was no clear correlation between ALCAM and MB histologic subtypes in the large cohort included in the Cavalli-763 MB dataset.

#2-13: There are several typos and grammatical errors. For example: “Table1: Adult (≥ 16 yeas)” instead of years; The correct spelling is “partially” not “partial” in the staining pattern description; In page 5 line 88 “many names” is used instead of “alias”; “Subdivision” is used instead of “subgroups”).

Response: We have corrected the suggested points and re-proofread the entire manuscript for other corresponding changes that were needed.
