# Supplementary material for: Activated leukocyte cell adhesion molecule expression correlates with the WNT subgroup in medulloblastoma and is involved in regulating tumor cell proliferation and invasion
Source: PLoS One. 2020 Dec 3;15(12):e0243272. doi: 10.1371/journal.pone.0243272 (PMC7714159; doi:10.1371/journal.pone.0243272)
Supplement: Attachment — Submitted filename: Response to Reviewers.docx [file pone.0243272.s004.docx]

Reviewer #2

**Major Comments:**

#2-1:

The MB FFPE cohort conformed by 36 cases used in the study is far too small to support the conclusions. The authors should prove their findings in a larger cohort. The strength of the preliminary data (IHC of 36 samples) is not sufficient to support the rationale of ALCAM as a WNT-related biomarker.

Response:

As pointed out, a validation cohort is essential for the biomarker study as our sample size was too small. We were only able to demonstrate an association between a strong expression of ALCAM and the WNT-subgroup in MB and not prove it as a biomarker. Accordingly, we revised our manuscript and ensured we did not define ALCAM as a WNT-related biomarker. Additionally, we included the aforementioned point as a limitation of our study, in the Discussion section of the revised manuscript. In addition, to expand our cohort as much as possible, we analyzed nine additional MB cases after the extension of the case enrollment period and the addition of another collaborating institution. Among the additional MB cases, the association between the WNT subgroup and the expression of ALCAM was similar to that in the original cohort in our study. One case was a WNT subgroup case and ALCAM positive, while eight cases were non-WNT and were ALCAM negative. These new cases and their details were included in the revised manuscript; Table 1, and Table 2 were updated accordingly. The ROC curve was also revised accordingly (Fig 1B).

#2-2:

Biomarker studies are not acceptable if authors do not validate their results in an independent cohort using the same technique. In this case, the independent validation the authors provide is an in silico validation (Cavalli et al. Cancer Cell 2017) at the gene expression level. Authors do not validate their results at the protein level using the IHC technique or other method to detect protein levels. The in silico analysis suggests a tendency but not a validation itself.

Response:

As mentioned above, in this study were only able to demonstrate an association between a strong expression of ALCAM and the WNT-subgroup in MB and not prove it as a biomarker. We used IHC to evaluate the expression of ALCAM in our cohort; the large cohorts of the Cavalli-763 MB dataset we used for verification referred to mRNA gene expression. To further strengthen the correlation between ALCAM protein levels showed by IHC and ALCAM mRNA gene expression levels, we examined the eight additional MB cases included in the revised manuscript (one ALCAM-positive and seven ALCAM-negative cases) using RT-qPCR. The correlation between ALCAM protein levels based on IHC and ALCAM mRNA expression revealed by RT-qPCR was statistically significant (*P* = 0.0237 to *P* = 0.0017; Fig 1A). The manuscript was revised accordingly.

#2-3:

The authors propose ALCAM IHC as an additional WNT-related biomarker to β-catenin to improve the reliability of diagnosis. However, ALCAM IHC is positive in six WNT-MB (n=6/6, 100% of all WNT analyzed) and presents an inconsistent positive result in one SHH (n=1/5, 20% of all SHH analyzed). This is not acceptable in a small cohort of 36 samples if the authors pretend to propose ALCAM as a biomarker.

Response:

As noted above, we have revised the Discussion section and did not define the expression of ALCAM as a potential biomarker. With respect to the cohort size, again we have included as many additional cases as possible. IHC staining of ALCAM was positive in all seven of the WNT-MB cases (n = 7/7, 100%) and one of the SHH-MB cases analyzed (n = 1/8, 12.5%). The expanded results were added to the revised manuscript.

#2-4:

The IHC training cohort of 36 FFPE samples is small and not fully characterized:

a. 2 NA Molecular subgroup

b. 5 NA CCNB1 status

Response:

a. 2 NA Molecular subgroup

Due to problems with some of the samples, Nanostring analysis could not be performed and the molecular subgroup was not determined in 2 cases (MB4, MB10). However, these cases definitely belonged to non-WNT subgroups: the *CTNNB1* mutation was not confirmed and β-catenin nuclear staining was negative. Furthermore, the pathological type of case MB4 was desmoplastic/nodular, suggesting that it was of the SHH subgroup. This information is provided in Table 2 of the revised manuscript.

b. 5 NA CCNB1 status

We have performed additional analysis regarding the *CTNNB1* status for all cases.

**Minor Comments:**

#2-5:

There is much more updated MB literature than the referenced: Northcott, P.A., Robinson, G.W., Kratz, C.P. et al. Medulloblastoma. Nat Rev Dis Primers 5, 11 (2019). https://doi.org/10.1038/s41572-019-0063-6

Response:

As suggested, we have added this citation to the Introduction section of the revised manuscript.

#2-6:

Authors declare that ALCAM depletion is associated with a more invasive tumour cells phenotype in vivo. This could be confirmed by a transwell migration assay analysis.

Response:

As per the reviewer’s suggestion, we performed transwell assays using Daoy cells. There was no remarkable difference in the invasion ability of ALCAM-depleted Daoy cells in this *in vitro* assay compared with that of Daoy control cells (*P* = 0.581; Fig 4E). We considered that the increased invasiveness exhibited by ALCAM-depleted Daoy cells in the context of meningeal dissemination *in vivo* may be related to other factors that are not implicated in invasion in the context of *in vitro* transwell assays. The results of the transwell assay were added to the revised manuscript.

#2-7:

Figure 2H: ALCAM was “weakly” detected in normal cerebellum in granular layer and white matter by IHC. The partially IHC staining of ALCAM in normal tissues should be further explored in normal cerebellum as well as brain if authors propose ALCAM as a possible WNT-related biomarker.

Response:

ALCAM was slightly expressed in the granular layer of the cerebellum, as shown in the manuscript. Of note, Allmendinger et al. (2012) have previously reported that ALCAM is expressed in normal cerebral tissue, e.g. in the hippocampus and basal ganglia, as well as in reactive glial cells. We have used immunohistochemistry to evaluate the expression of ALCAM in representative tumor areas in the current study. We have revised the Discussion section of the manuscript to include a description of the expression of ALCAM in normal brain tissues.

#2-8:

Positive ALCAM IHC staining of one of the five SHH tumor samples included in the study as well as high ALCAM mRNA levels in SHH cell lines (DAOY and ONS-76) should be declared and further investigated in order to understand the role of ALCAM in MB.

Response:

As suggested, we have included this point in the Discussion section of the revised manuscript.
