# Supplementary material for: Activated leukocyte cell adhesion molecule expression correlates with the WNT subgroup in medulloblastoma and is involved in regulating tumor cell proliferation and invasion
Source: PLoS One. 2020 Dec 3;15(12):e0243272. doi: 10.1371/journal.pone.0243272 (PMC7714159; doi:10.1371/journal.pone.0243272)
Supplement: Attachment — Submitted filename: Response to Reviewers.docx [file pone.0243272.s006.docx]

Reviewer #2

**Minor Comments:**

Authors need to clarify the finality and the description of the ROC curve analysis. It is not clear if the ROC curve analysis is meant to select ALCAM IHC positivity cut-point neither how many MB samples are used or their subgroup. Authors must remove the word “diagnosis” from the manuscript in line 325 “To evaluate the reliability of the WNT subgroup diagnosis of MB using ALCAM” as it is misleading.

Response:

Thank you for your valuable comment. As pointed out, we have removed the word “diagnosis” from the sentence to avoid confusion. As suggested, we have modified the description of the ROC curve analysis and added information on the number of MB cases and the subgroup. In the Methods section of the manuscript, the description of the ROC curve analysis was modified and moved from the sub-section “statistical analysis” to “Immunohistochemistry”.
