# Supplementary material for: Activated leukocyte cell adhesion molecule expression correlates with the WNT subgroup in medulloblastoma and is involved in regulating tumor cell proliferation and invasion
Source: PLoS One. 2020 Dec 3;15(12):e0243272. doi: 10.1371/journal.pone.0243272 (PMC7714159; doi:10.1371/journal.pone.0243272)
Supplement: Peer review history

# Peer review: Decision Letter 0

**DOI:** 10.1371/journal.pone.0243272.r001

**Authors:** Masaru Katoh

1 Aug 2019

PONE-D-19-17600

The expression of activated leukocyte cell adhesion molecule correlates with the WNT subgroup in medulloblastoma and is involved in the regulation of tumor cell invasion

PLOS ONE

Dear Dr Kijima,

Thank you for submitting your manuscript to PLOS ONE. After careful consideration, we feel that it has merit but does not fully meet PLOS ONE’s publication criteria as it currently stands. Therefore, we invite you to submit a revised version of the manuscript that addresses the points raised during the review process.

(1) ALCAM overexpression experiments 

(2) Presentation of ROC curve and description of cut-off value for ALCAM immunohistochemistry

(3) Discussion related to ALCAM immunohistochemistry, such as (i) ALCAM immunostaining pattern, (ii) ALCAM-positive and nuclear ß-catenin-negative cases, (iii) ALCAM mRNA expression levels in medulloblastoma subgroups

(4) Discussion of weak points in this report, such as (i) small sample size and (ii) lack of validation study.  In addition, the authors need to discuss statistical power of a clinical study based on 36 cases of medulloblastoma patients.

(5) Language edition

(6) Other issues pointed out by Reviewers

We would appreciate receiving your revised manuscript by Sep 15 2019 11:59PM. When you are ready to submit your revision, log on to https://www.editorialmanager.com/pone/ and select the 'Submissions Needing Revision' folder to locate your manuscript file.

If you would like to make changes to your financial disclosure, please include your updated statement in your cover letter.

To enhance the reproducibility of your results, we recommend that if applicable you deposit your laboratory protocols in protocols.io, where a protocol can be assigned its own identifier (DOI) such that it can be cited independently in the future. For instructions see: http://journals.plos.org/plosone/s/submission-guidelines#loc-laboratory-protocols

Please include the following items when submitting your revised manuscript:

- A rebuttal letter that responds to each point raised by the academic editor and reviewer(s). This letter should be uploaded as separate file and labeled 'Response to Reviewers'.
- A marked-up copy of your manuscript that highlights changes made to the original version. This file should be uploaded as separate file and labeled 'Revised Manuscript with Track Changes'.
- An unmarked version of your revised paper without tracked changes. This file should be uploaded as separate file and labeled 'Manuscript'.

Please note while forming your response, if your article is accepted, you may have the opportunity to make the peer review history publicly available. The record will include editor decision letters (with reviews) and your responses to reviewer comments. If eligible, we will contact you to opt in or out.

We look forward to receiving your revised manuscript.

Kind regards,

Masaru Katoh, M.D., Ph.D.

Academic Editor

PLOS ONE

Journal Requirements:

1. When submitting your revision, we need you to address these additional requirements.

Please ensure that your manuscript meets PLOS ONE's style requirements, including those for file naming. The PLOS ONE style templates can be found at

http://www.journals.plos.org/plosone/s/file?id=wjVg/PLOSOne_formatting_sample_main_body.pdf and http://www.journals.plos.org/plosone/s/file?id=ba62/PLOSOne_formatting_sample_title_authors_affiliations.pdf

2. 1) To comply with PLOS ONE submissions requirements, in your Methods section, please provide additional information regarding the experiments involving animals and ensure you have included details on (1) methods of sacrifice, (2) methods of anesthesia and/or analgesia, and (3) efforts to alleviate suffering. Please also ensure that you have described the human endpoints ("severe neurological symptoms") in detail, what were the symptoms that would warrant immediate sacrifice?

2) Thank you for including the statement "For all cases, either written informed consent was obtained or its requirement was waived by a public announcement" regarding the case files analysed in this study. It is not clear what "public announcement" means in this context; was consent waived by an ethics committee or IRB? Please ensure that you have discussed whether all data were fully anonymized before you accessed them and/or whether the IRB or ethics committee waived the requirement for informed consent.

[Note: HTML markup is below. Please do not edit.]

Reviewers' comments:

Reviewer's Responses to Questions

**Comments to the Author**

1. Is the manuscript technically sound, and do the data support the conclusions?

The manuscript must describe a technically sound piece of scientific research with data that supports the conclusions. Experiments must have been conducted rigorously, with appropriate controls, replication, and sample sizes. The conclusions must be drawn appropriately based on the data presented. 

Reviewer #1: Partly

Reviewer #2: No

**********

2. Has the statistical analysis been performed appropriately and rigorously? 

Reviewer #1: Yes

Reviewer #2: No

**********

3. Have the authors made all data underlying the findings in their manuscript fully available?

The PLOS Data policy requires authors to make all data underlying the findings described in their manuscript fully available without restriction, with rare exception (please refer to the Data Availability Statement in the manuscript PDF file). The data should be provided as part of the manuscript or its supporting information, or deposited to a public repository. For example, in addition to summary statistics, the data points behind means, medians and variance measures should be available. If there are restrictions on publicly sharing data—e.g. participant privacy or use of data from a third party—those must be specified.

Reviewer #1: Yes

Reviewer #2: Yes

**********

4. Is the manuscript presented in an intelligible fashion and written in standard English?

PLOS ONE does not copyedit accepted manuscripts, so the language in submitted articles must be clear, correct, and unambiguous. Any typographical or grammatical errors should be corrected at revision, so please note any specific errors here.

Reviewer #1: Yes

Reviewer #2: No

**********

5. Review Comments to the Author

Please use the space provided to explain your answers to the questions above. You may also include additional comments for the author, including concerns about dual publication, research ethics, or publication ethics. (Please upload your review as an attachment if it exceeds 20,000 characters)

Reviewer #1: The Authors analyzed deeply the relationship between ALCAM expression and both cell proliferation and migration. There are few criticisms: they didn’t evaluated the effect of an overexpression induced in cell lines (mainly in the cell lines exhibiting a low expression level of ALCAM, D341, and D283) which should show an increasing rate of cell proliferation and migration (D341, and D28 proliferation and migration rate in normal conditions are not described). Moreover, it is well known that Daoy cell line exhibits mutated p53 which is tightly related to beta catenin pathway. The Authors could analyze the p53 mutation status also in Daoy induced MB (p53 wild type or not) to give a hypothesis to explain the different results among different experimental conditions (“in vitro” and ”in vivo”)

Reviewer #2: The aim of the present study was to reveal the functional role and significance of ALCAM expression in Medulloblastoma (MB). In the first part of the results, the authors carried out several functional in vitro and in vivo assays by silencing ALCAM using RNA interference in order to unveil the function of ALCAM in MB. In the second part of the study, the authors provide new information about ALCAM expression correlation with MB molecular and histological subtypes in a MB FFPE cohort conformed by 39 patients. As a conclusion, authors propose ALCAM as a novel MB WNT biomarker.

Reviewers Concerns:

- Authors should declare that the clinical stratification of MB tumors is stated at the 2016 Consensus Paper (Ramaswamy, V et al. Acta Neuropathol (2016) 131:821–831).

- The article seems to report two well-differentiated studies aimed at: i) the functional role of ALCAM in MB models and ii) the potential use of ALCAM expression as an IHC biomarker in MB, however, both studies are incomplete. Additional analyses are necessary to clarify the role of ALCAM as well as to propose ALCAM as a potential biomarker for the MB - WNT subgroup.

- Review of the Functional Analyses:

o Author should explain why MB cell lines were cultured in different medium and FBSi conditions. This could be a source of variability.

o Results reported in Fig1H show differences of proliferation that are hardly appreciable for the ONS-76 cell lines at 48h. Author do not specify the significance of the *** symbol.

o Authors should perform additional assays using different MB cell lines, in order to explore further the effect of ALCAM depletion on migration/invasion, and clarify the contradictory findings observed between in vitro and in vivo assays.

- Review of the IHC analysis:

o The contents of the ALCAM expression analysis section should be placed before the functional part, making it easier to understand the rational of the study and to follow the results.

o The MB FFPE cohort conformed by 36 cases used in the study is far too small to support the conclusions and includes both pediatric and adult MB cases. Only six of these are WNT MBs. The authors should prove their findings in a larger cohort.

o Table 1, ALCAM positive staining was observed in two cases (one SHH) with negative nuclear beta-catenin expression. This is critical for the study and questions the ALCAM staining as a reliable marker. Authors must verify the presence of CTNNB1 mutation.

o In addition, no biomarker studies are acceptable if the authors do not validate their results in an independent cohort.

o MB is rarely diagnosed in adults, whereas MB is the most common malignant pediatric brain tumor. However, four of the six MB WNT cases included in the FFPE cohort were adults, only two children. Adult MB is distinct from childhood MB with clear differences in the molecular variants and clinical evolution, and should thus be analysed separately. Authors do not address this issue. The cohort is biased and does not fully represent the MB WNT subgroup.

o Authors describe the IHC pattern of ALCAM in WNT-MB samples predominantly in the cytoplasm, but also the presence of some staining in the cell membrane. If authors propose the use of ALCAM as a MB-WNT biomarker, the IHC staining pattern must be described accurately (see Mezzanzanica, D et al. Clin Cancer Res 2008;14(6)). IHC consistent controls should be reported.

o Studies that propose possible biomarkers should include analyses such as ROC curves to demonstrate the reliability and validity of the results.

o The correlation between ALCAM IHC staining and ALCAM mRNA expression by qPCR was performed in a limited number of samples: 3 positive vs 6 negative for ALCAM IHC.

o The ALCAM mRNA validation in Cavalli microarray cohort (n=763) showed a large variability of ALCAM expression within subgroups and overlap across MB subgroups. ALCAM mRNA expression in MB subgroups has low specificity based on the presented results.

o Authors did not assess the correlation between ALCAM expression and the MB histological subtypes as the cohort does not has a fully representation of all histological MB subtypes. Authors should enlarge the cohort all MB histological subtypes in order to obtain robust conclusions.

o The IHC cut-off values for subdivision must be more restrictive in a biomarker study to be reliable.

- The results are insufficient to support the exposed conclusions.

- There are several typos and grammatical errors. For example: “Table1: Adult (≥ 16 yeas)” instead of years; The correct spelling is “partially” not “partial” in the staining pattern description; In page 5 line 88 “many names” is used instead of “alias”; “Subdivision” is used instead of “subgroups”).

**********

6. PLOS authors have the option to publish the peer review history of their article (what does this mean?). If published, this will include your full peer review and any attached files.

If you choose “no”, your identity will remain anonymous but your review may still be made public.

**Do you want your identity to be public for this peer review?** For information about this choice, including consent withdrawal, please see our Privacy Policy.

Reviewer #1: No

Reviewer #2: No

[NOTE: If reviewer comments were submitted as an attachment file, they will be attached to this email and accessible via the submission site. Please log into your account, locate the manuscript record, and check for the action link "View Attachments". If this link does not appear, there are no attachment files to be viewed.]

While revising your submission, please upload your figure files to the Preflight Analysis and Conversion Engine (PACE) digital diagnostic tool, https://pacev2.apexcovantage.com/. PACE helps ensure that figures meet PLOS requirements. To use PACE, you must first register as a user. Registration is free. Then, login and navigate to the UPLOAD tab, where you will find detailed instructions on how to use the tool. If you encounter any issues or have any questions when using PACE, please email us at figures@plos.org. Please note that Supporting Information files do not need this step.

Attachment Submitted filename: ReviewCommentsAuthors_PONE-D-19-17600.pdf Click here for additional data file.

# Author comment: Author response to Decision Letter 0

**DOI:** 10.1371/journal.pone.0243272.r002

4 Feb 2020

Editor

(1) ALCAM overexpression experiments 

Response: Related to #1-1, we have performed ALCAM overexpression experiments using the D341 cell line and showed that ALCAM promotes cell proliferation (Fig 3J and 3K). 

(2) Presentation of ROC curve and description of cut-off value for ALCAM immunohistochemistry

Response: Related to #2-10, we have created ROC curves and evaluated the diagnostic performances of ALCAM Immunohistochemical staining and have identified optimal cut-off points for the diagnosis of the WNT molecular subgroup (Fig 1B).

(3) Discussion related to ALCAM immunohistochemistry, such as (i) ALCAM immunostaining pattern, (ii) ALCAM-positive and nuclear ß-catenin-negative cases, (iii) ALCAM mRNA expression levels in medulloblastoma subgroups

Response: Related to #2-9, #2-11, and #2-12, we described the immunostaining patterns in all ALCAM-positive staining cases in Table 2. Moreover, we examined the histologic subtype and age group of MB with respect to the expression of ALCAM (Fig 1D and 1E).

(4) Discussion of weak points in this report, such as (i) small sample size and (ii) lack of validation study. In addition, the authors need to discuss statistical power of a clinical study based on 36 cases of medulloblastoma patients.

Response: Related to #2-6, we have described these as limitations in the Discussion section.

(5) Language edition

Response: Related to #2-13, we have proofread the entire manuscript.

(6) Other issues pointed out by Reviewers

Response: We have responded to all reviewer questions as described below.

Reviewer #1

#1-1: They didn’t evaluated the effect of an overexpression induced in cell lines (mainly in the cell lines exhibiting a low expression level of ALCAM, D341, and D283) which should show an increasing rate of cell proliferation and migration (D341, and D28 proliferation and migration rate in normal conditions are not described). 

Response: The ALCAM overexpression experiments using the D341 cell line showed that enhanced ALCAM expression increased cell proliferation. D341 cells migration could not be evaluated due to the feature that D341 cells grow in suspension and not as adherent monolayers. This has been noted in the revised manuscript. 

#1-2: It is well known that Daoy cell line exhibits mutated p53 which is tightly related to beta catenin pathway. The Authors could analyze the p53 mutation status also in Daoy induced MB (p53 wild type or not) to give a hypothesis to explain the different results among different experimental conditions (“in vitro” and ”in vivo”) 

Response: We analyzed the TP53 gene mutation status both in vitro in Daoy cell lines (shControl, shALCAM1, and shALCAM2) and in vivo in Daoy tumor tissues (shControl and shALCAM1). Sanger sequencing analysis of the Exon 2-11 region detected a common c.725G>T: p.C242F homozygous mutation in the TP53 gene of all samples. This was consistent with the known mutation reported in Daoy line (Saylors et al., 1991).

Reviewer #2

#2-1: Authors should declare that the clinical stratification of MB tumors is stated at the 2016 Consensus Paper (Ramaswamy, V et al. Acta Neuropathol (2016) 

131V821–831).

Response: As suggested, we have added this to the Introduction of the revised manuscript and provided the proper in-text citation of this consensus paper.

#2-2: Author should explain why MB cell lines were cultured in different medium and FBSi conditions. This could be a source of variability. 

Response: We used the media and FBS conditions recommended for each cell line. Culturing may not be successful for a particular cell line when conditions are changed.

#2-3: Results reported in Fig1H show differences of proliferation that are hardly appreciable for the ONS-76 cell lines at 48h. Author do not specify the significance of the *** symbol.

Response: We apologize for the error in defining the sign of the significance. We revised the description from "*: P <0.05; **: P <0.0001" to "*: P < 0.05; **: P < 0.01; ***: P < 0.0001" and revised the figure as well.

#2-4: Authors should perform additional assays using different MB cell lines, in order to explore further the effect of ALCAM depletion on migration/invasion, and clarify the contradictory findings observed between in vitro and in vivo assays.

Response: We performed an ALCAM overexpression experiment using the D341 cell line. Enhanced ALCAM expression increased cell proliferation. An orthotopic mouse model using ONS-76 cells was also evaluated, but meningeal dissemination was not well formed.

#2-5: The contents of the ALCAM expression analysis section should be placed before the functional part, making it easier to understand the rational of the study and to follow the results.

Response: As the reviewer suggested, we have restructured the appropriate sections in the revised manuscript.

#2-6: The MB FFPE cohort conformed by 36 cases used in the study is far too small to support the conclusions and includes both pediatric and adult MB cases. Only six of these are WNT MBs. The authors should prove their findings in a larger cohort.

&

In addition, no biomarker studies are acceptable if the authors do not validate their results in an independent cohort.

Response: Due to sample limitations, our cohort size was relatively small and our study lacked a validation component using ALCAM immunohistochemical staining. However, analysis from a large cohort (the Cavalli-763 MB dataset of the R2: Genomics Analysis and Visualization Platform) based on gene expression analysis showed a strong correlation between ALCAM expression and the WNT subgroup of MB. We have included the small sample size our cohort as a limitation of our study in the Discussion of the revised manuscript. 

#2-7: Table 1, ALCAM positive staining was observed in two cases (one SHH) with negative nuclear beta-catenin expression. This is critical for the study and questions the ALCAM staining as a reliable marker. Authors must verify the presence of CTNNB1 mutation.

Response: As shown in Table 1, we have verified CTNNB1 mutations in most of the cases used in our study. 

#2-8: MB is rarely diagnosed in adults, whereas MB is the most common malignant pediatric brain tumor. However, four of the six MB WNT cases included in the FFPE cohort were adults, only two children. Adult MB is distinct from childhood MB with clear differences in the molecular variants and clinical evolution, and should thus be analysed separately. Authors do not address this issue. The cohort is biased and does not fully represent the MB WNT subgroup.

Response: MB is pediatric brain tumor, but the age distribution differs for each molecular subgroup. Adults (> 16 y) are not uncommon in WNT, SHH, and Group 4 subgroups of MB. In the WNT subgroup, infant cases (< 4 y) are rare and cases of children (4-16 y) and adults (>16 years) occur at a 2:1 ratio (Kool et al., 2012 and Taylor et al., 2012). In our cohort, the cases of the WNT subgroup included two children (6 and 7 years old) and four adults (16, 17, 18, and 20 years old) according to the original age distribution definition (Adult: ≥ 16 years, child: < 16 and ≥ 3 years, and infant: < 3 years). We have changed the definition of age distribution in the revised manuscript to be consistent with other cohorts (Adult: > 16 years, child: 4-16 years, infant: < 4 years). The cases of the WNT subgroup now included three children (6, 7, and 16 years old) and three adults (17, 18, and 20 years old). There was no significant difference in the age distribution of the other cohorts and both the child and adult cases were positive for ALCAM immunohistochemical staining.

#2-9: Authors describe the IHC pattern of ALCAM in WNT-MB samples predominantly in the cytoplasm, but also the presence of some staining in the cell membrane. If authors propose the use of ALCAM as a MB-WNT biomarker, the IHC staining pattern must be described accurately (see Mezzanzanica, D et al. Clin Cancer Res 2008;14(6)). IHC consistent controls should be reported. 

Response: As suggested, we have added the information regarding ALCAM IHC pattern to Table 2 in the revised manuscript.

#2-10: Studies that propose possible biomarkers should include analyses such as ROC curves to demonstrate the reliability and validity of the results.

&

The IHC cut-off values for subdivision must be more restrictive in a biomarker study to be reliable.

Response: We have included the accuracy of the WNT subgroup diagnosis by ALCAM immunohistochemical staining and the optimal cutoff value according to ROC curve analysis.

#2-11: The ALCAM mRNA validation in Cavalli microarray cohort (n=763) showed a large variability of ALCAM expression within subgroups and overlap across MB subgroups. ALCAM mRNA expression in MB subgroups has low specificity based on the presented results.

Response: As pointed out, in a large cohort used for validation (the Cavalli-763 MB dataset), ALCAM expression in some cases exhibited overlap between MB subgroups. As a result of additional analysis, it became clear that the cases where ALCAM expression was low in the WNT subgroup, the patients were more than 21 years old. We have described this in the Results of the revised manuscript and added it as a limitation of our study.

#2-12: Authors did not assess the correlation between ALCAM expression and the MB histological subtypes as the cohort does not has a fully representation of all histological MB subtypes. Authors should enlarge the cohort all MB histological subtypes in order to obtain robust conclusions.

Response: In our small cohort, the histologic subtype was biased and difficult to evaluate. However, there was no clear correlation between ALCAM and MB histologic subtypes in the large cohort included in the Cavalli-763 MB dataset.

#2-13: There are several typos and grammatical errors. For example: “Table1: Adult (≥ 16 yeas)” instead of years; The correct spelling is “partially” not “partial” in the staining pattern description; In page 5 line 88 “many names” is used instead of “alias”; “Subdivision” is used instead of “subgroups”). 

Response: We have corrected the suggested points and re-proofread the entire manuscript for other corresponding changes that were needed.

Attachment Submitted filename: Response_to_Reviewers.docx Click here for additional data file.

# Peer review: Decision Letter 1

**DOI:** 10.1371/journal.pone.0243272.r003

**Authors:** Javier S Castresana

6 Mar 2020

PONE-D-19-17600R1

The expression of activated leukocyte cell adhesion molecule correlates with the WNT subgroup in medulloblastoma and is involved in the regulation of tumor cell invasion

PLOS ONE

Dear Dr Kijima,

Thank you for submitting your manuscript to PLOS ONE. After careful consideration, we feel that it has merit but does not fully meet PLOS ONE’s publication criteria as it currently stands. Therefore, we invite you to submit a revised version of the manuscript that addresses the points raised during the review process.

We would appreciate receiving your revised manuscript by Apr 20 2020 11:59PM. When you are ready to submit your revision, log on to https://www.editorialmanager.com/pone/ and select the 'Submissions Needing Revision' folder to locate your manuscript file.

If you would like to make changes to your financial disclosure, please include your updated statement in your cover letter.

To enhance the reproducibility of your results, we recommend that if applicable you deposit your laboratory protocols in protocols.io, where a protocol can be assigned its own identifier (DOI) such that it can be cited independently in the future. For instructions see: http://journals.plos.org/plosone/s/submission-guidelines#loc-laboratory-protocols

Please include the following items when submitting your revised manuscript:

- A rebuttal letter that responds to each point raised by the academic editor and reviewer(s). This letter should be uploaded as separate file and labeled 'Response to Reviewers'.
- A marked-up copy of your manuscript that highlights changes made to the original version. This file should be uploaded as separate file and labeled 'Revised Manuscript with Track Changes'.
- An unmarked version of your revised paper without tracked changes. This file should be uploaded as separate file and labeled 'Manuscript'.

Please note while forming your response, if your article is accepted, you may have the opportunity to make the peer review history publicly available. The record will include editor decision letters (with reviews) and your responses to reviewer comments. If eligible, we will contact you to opt in or out.

We look forward to receiving your revised manuscript.

Kind regards,

Javier S Castresana

Academic Editor

PLOS ONE

Additional Editor Comments (if provided):

Please, try to answer to the comments made by reviewer number 2. If not possible to do it experimentally, do it at least by an explanation to all her comments.

[Note: HTML markup is below. Please do not edit.]

Reviewers' comments:

Reviewer's Responses to Questions

**Comments to the Author**

1. If the authors have adequately addressed your comments raised in a previous round of review and you feel that this manuscript is now acceptable for publication, you may indicate that here to bypass the “Comments to the Author” section, enter your conflict of interest statement in the “Confidential to Editor” section, and submit your "Accept" recommendation.

Reviewer #1: All comments have been addressed

Reviewer #2: (No Response)

**********

2. Is the manuscript technically sound, and do the data support the conclusions?

The manuscript must describe a technically sound piece of scientific research with data that supports the conclusions. Experiments must have been conducted rigorously, with appropriate controls, replication, and sample sizes. The conclusions must be drawn appropriately based on the data presented. 

Reviewer #1: Yes

Reviewer #2: Partly

**********

3. Has the statistical analysis been performed appropriately and rigorously? 

Reviewer #1: Yes

Reviewer #2: No

**********

4. Have the authors made all data underlying the findings in their manuscript fully available?

The PLOS Data policy requires authors to make all data underlying the findings described in their manuscript fully available without restriction, with rare exception (please refer to the Data Availability Statement in the manuscript PDF file). The data should be provided as part of the manuscript or its supporting information, or deposited to a public repository. For example, in addition to summary statistics, the data points behind means, medians and variance measures should be available. If there are restrictions on publicly sharing data—e.g. participant privacy or use of data from a third party—those must be specified.

Reviewer #1: Yes

Reviewer #2: Yes

**********

5. Is the manuscript presented in an intelligible fashion and written in standard English?

PLOS ONE does not copyedit accepted manuscripts, so the language in submitted articles must be clear, correct, and unambiguous. Any typographical or grammatical errors should be corrected at revision, so please note any specific errors here.

Reviewer #1: Yes

Reviewer #2: Yes

**********

6. Review Comments to the Author

Please use the space provided to explain your answers to the questions above. You may also include additional comments for the author, including concerns about dual publication, research ethics, or publication ethics. (Please upload your review as an attachment if it exceeds 20,000 characters)

Reviewer #1: The paper has been modified following the reviewer suggestions and criticisms and In the present revised form is now ready to publish

Reviewer #2: Activated leukocyte cell adhesion molecule expression correlates with the WNT subgroup in medulloblastoma and is involved in regulating tumor cell proliferation and invasion

Takamune Achiha, Noriyuki Kijima, Yoshinori Kodama, Naoki Kagawa, Manabu Kinoshita, Yasunori Fujimoto, Masahito Nonaka, Namiko Nishida, Takumi Yamanaka, Atsuko Harada, Kanji Mori, Junya Fukai, Naohiro Tsuyuguchi, Takehiro Uda, Kenichi Ishibashi, Yusuke Tomogane, Daisuke Sakamoto, Tomoko Shofuda, Ema Yoshioka, Daisuke Kanematsu, Masayuki Mano, Betty Luu, Michael D. Taylor, Yonehiro Kanemura, Haruhiko Kishima.

The aim of the present study was to reveal the functional role and significance of ALCAM expression in Medulloblastoma (MB). The authors provide new information of ALCAM protein levels analysed by IHC in a MB FFPE cohort conformed by 36 patient and performed an in silico correlation between ALCAM gene expression and the MB molecular and histological subtypes as well as patient age’s in a large MB cohort from the R2 genomics Platform (Cavalli et al. Cancer Cell 2017). In the second part of the study, the authors carried out several functional in vitro and in vivo assays by silencing ALCAM using RNA interference in order to unveil the function of ALCAM in MB. As a conclusion, authors offer ALCAM as a novel MB WNT biomarker.

Reviewer Concerns:

There has been a qualitative improvement in the manuscript compared to the previous version. The authors have restructured the article and now the results are easier to follow, however, the results are still insufficient to support the conclusions presented. The article is attractive and unveils an interesting role of ALCAM in MB-WNT subgroup, but it has to be reported as a study of the MB underlying biology, not as a biomarker study.

Major Comments:

- The MB FFPE cohort conformed by 36 cases used in the study is far too small to support the conclusions. The authors should prove their findings in a larger cohort. The strength of the preliminary data (IHC of 36 samples) is not sufficient to support the rationale of ALCAM as a WNT-related biomarker.

- Biomarker studies are not acceptable if authors do not validate their results in an independent cohort using the same technique. In this case, the independent validation the authors provide is an in silico validation (Cavalli et al. Cancer Cell 2017) at the gene expression level. Authors do not validate their results at the protein level using the IHC technique or other method to detect protein levels. The in silico analysis suggests a tendency but not a validation itself.

- The authors propose ALCAM IHC as an additional WNT-related biomarker to β-catenin to improve the reliability of diagnosis. However, ALCAM IHC is positive in six WNT-MB (n=6/6, 100% of all WNT analyzed) and presents an inconsistent positive result in one SHH (n=1/5, 20% of all SHH analyzed). This is not acceptable in a small cohort of 36 samples if the authors pretend to propose ALCAM as a biomarker.

- The IHC training cohort of 36 FFPE samples is small and not fully characterized:

a. 2 NA Molecular subgroup

b. 5 NA CCNB1 status

Minor Comments

- There is much more updated MB literature than the referenced: Northcott, P.A., Robinson, G.W., Kratz, C.P. et al. Medulloblastoma. Nat Rev Dis Primers 5, 11 (2019). https://doi.org/10.1038/s41572-019-0063-6

- Authors declare that ALCAM depletion is associated with a more invasive tumour cells phenotype in vivo. This could be confirmed by a transwell migration assay analysis.

- Figure 2H: ALCAM was “weakly” detected in normal cerebellum in granular layer and white matter by IHC. The partially IHC staining of ALCAM in normal tissues should be further explored in normal cerebellum as well as brain if authors propose ALCAM as a possible WNT-related biomarker.

- Positive ALCAM IHC staining of one of the five SHH tumor samples included in the study as well as high ALCAM mRNA levels in SHH cell lines (DAOY and ONS-76) should be declared and further investigated in order to understand the role of ALCAM in MB.

**********

7. PLOS authors have the option to publish the peer review history of their article (what does this mean?). If published, this will include your full peer review and any attached files.

If you choose “no”, your identity will remain anonymous but your review may still be made public.

**Do you want your identity to be public for this peer review?** For information about this choice, including consent withdrawal, please see our Privacy Policy.

Reviewer #1: No

Reviewer #2: No

[NOTE: If reviewer comments were submitted as an attachment file, they will be attached to this email and accessible via the submission site. Please log into your account, locate the manuscript record, and check for the action link "View Attachments". If this link does not appear, there are no attachment files to be viewed.]

While revising your submission, please upload your figure files to the Preflight Analysis and Conversion Engine (PACE) digital diagnostic tool, https://pacev2.apexcovantage.com/. PACE helps ensure that figures meet PLOS requirements. To use PACE, you must first register as a user. Registration is free. Then, login and navigate to the UPLOAD tab, where you will find detailed instructions on how to use the tool. If you encounter any issues or have any questions when using PACE, please email us at figures@plos.org. Please note that Supporting Information files do not need this step.

Attachment Submitted filename: ReviewerAttachments.pdf Click here for additional data file.

# Author comment: Author response to Decision Letter 1

**DOI:** 10.1371/journal.pone.0243272.r004

27 Oct 2020

Reviewer #2

Major Comments:

#2-1:

The MB FFPE cohort conformed by 36 cases used in the study is far too small to support the conclusions. The authors should prove their findings in a larger cohort. The strength of the preliminary data (IHC of 36 samples) is not sufficient to support the rationale of ALCAM as a WNT-related biomarker. 

Response:

As pointed out, a validation cohort is essential for the biomarker study as our sample size was too small. We were only able to demonstrate an association between a strong expression of ALCAM and the WNT-subgroup in MB and not prove it as a biomarker. Accordingly, we revised our manuscript and ensured we did not define ALCAM as a WNT-related biomarker. Additionally, we included the aforementioned point as a limitation of our study, in the Discussion section of the revised manuscript. In addition, to expand our cohort as much as possible, we analyzed nine additional MB cases after the extension of the case enrollment period and the addition of another collaborating institution. Among the additional MB cases, the association between the WNT subgroup and the expression of ALCAM was similar to that in the original cohort in our study. One case was a WNT subgroup case and ALCAM positive, while eight cases were non-WNT and were ALCAM negative. These new cases and their details were included in the revised manuscript; Table 1, and Table 2 were updated accordingly. The ROC curve was also revised accordingly (Fig 1B).

#2-2:

Biomarker studies are not acceptable if authors do not validate their results in an independent cohort using the same technique. In this case, the independent validation the authors provide is an in silico validation (Cavalli et al. Cancer Cell 2017) at the gene expression level. Authors do not validate their results at the protein level using the IHC technique or other method to detect protein levels. The in silico analysis suggests a tendency but not a validation itself.

Response:

As mentioned above, in this study were only able to demonstrate an association between a strong expression of ALCAM and the WNT-subgroup in MB and not prove it as a biomarker. We used IHC to evaluate the expression of ALCAM in our cohort; the large cohorts of the Cavalli-763 MB dataset we used for verification referred to mRNA gene expression. To further strengthen the correlation between ALCAM protein levels showed by IHC and ALCAM mRNA gene expression levels, we examined the eight additional MB cases included in the revised manuscript (one ALCAM-positive and seven ALCAM-negative cases) using RT-qPCR. The correlation between ALCAM protein levels based on IHC and ALCAM mRNA expression revealed by RT-qPCR was statistically significant (P = 0.0237 to P = 0.0017; Fig 1A). The manuscript was revised accordingly.

#2-3:

The authors propose ALCAM IHC as an additional WNT-related biomarker to β-catenin to improve the reliability of diagnosis. However, ALCAM IHC is positive in six WNT-MB (n=6/6, 100% of all WNT analyzed) and presents an inconsistent positive result in one SHH (n=1/5, 20% of all SHH analyzed). This is not acceptable in a small cohort of 36 samples if the authors pretend to propose ALCAM as a biomarker.

Response:

As noted above, we have revised the Discussion section and did not define the expression of ALCAM as a potential biomarker. With respect to the cohort size, again we have included as many additional cases as possible. IHC staining of ALCAM was positive in all seven of the WNT-MB cases (n = 7/7, 100%) and one of the SHH-MB cases analyzed (n = 1/8, 12.5%). The expanded results were added to the revised manuscript.

#2-4:

The IHC training cohort of 36 FFPE samples is small and not fully characterized:

a. 2 NA Molecular subgroup

b. 5 NA CCNB1 status

Response:

a. 2 NA Molecular subgroup

Due to problems with some of the samples, Nanostring analysis could not be performed and the molecular subgroup was not determined in 2 cases (MB4, MB10). However, these cases definitely belonged to non-WNT subgroups: the CTNNB1 mutation was not confirmed and β-catenin nuclear staining was negative. Furthermore, the pathological type of case MB4 was desmoplastic/nodular, suggesting that it was of the SHH subgroup. This information is provided in Table 2 of the revised manuscript.

b. 5 NA CCNB1 status

We have performed additional analysis regarding the CTNNB1 status for all cases.

Minor Comments:

#2-5:

There is much more updated MB literature than the referenced: Northcott, P.A., Robinson, G.W., Kratz, C.P. et al. Medulloblastoma. Nat Rev Dis Primers 5, 11 (2019). https://doi.org/10.1038/s41572-019-0063-6

Response:

As suggested, we have added this citation to the Introduction section of the revised manuscript.

#2-6:

Authors declare that ALCAM depletion is associated with a more invasive tumour cells phenotype in vivo. This could be confirmed by a transwell migration assay analysis.

Response:

As per the reviewer’s suggestion, we performed transwell assays using Daoy cells. There was no remarkable difference in the invasion ability of ALCAM-depleted Daoy cells in this in vitro assay compared with that of Daoy control cells (P = 0.581; Fig 4E). We considered that the increased invasiveness exhibited by ALCAM-depleted Daoy cells in the context of meningeal dissemination in vivo may be related to other factors that are not implicated in invasion in the context of in vitro transwell assays. The results of the transwell assay were added to the revised manuscript.

#2-7:

Figure 2H: ALCAM was “weakly” detected in normal cerebellum in granular layer and white matter by IHC. The partially IHC staining of ALCAM in normal tissues should be further explored in normal cerebellum as well as brain if authors propose ALCAM as a possible WNT-related biomarker.

Response:

ALCAM was slightly expressed in the granular layer of the cerebellum, as shown in the manuscript. Of note, Allmendinger et al. (2012) have previously reported that ALCAM is expressed in normal cerebral tissue, e.g. in the hippocampus and basal ganglia, as well as in reactive glial cells. We have used immunohistochemistry to evaluate the expression of ALCAM in representative tumor areas in the current study. We have revised the Discussion section of the manuscript to include a description of the expression of ALCAM in normal brain tissues.

#2-8:

Positive ALCAM IHC staining of one of the five SHH tumor samples included in the study as well as high ALCAM mRNA levels in SHH cell lines (DAOY and ONS-76) should be declared and further investigated in order to understand the role of ALCAM in MB.

Response:

As suggested, we have included this point in the Discussion section of the revised manuscript.

Attachment Submitted filename: Response to Reviewers.docx Click here for additional data file.

# Peer review: Decision Letter 2

**DOI:** 10.1371/journal.pone.0243272.r005

**Authors:** Javier S Castresana

12 Nov 2020

PONE-D-19-17600R2

Activated leukocyte cell adhesion molecule expression correlates with the WNT subgroup in medulloblastoma and is involved in regulating tumor cell proliferation and invasion

PLOS ONE

Dear Dr. Kijima,

Thank you for submitting your manuscript to PLOS ONE. After careful consideration, we feel that it has merit but does not fully meet PLOS ONE’s publication criteria as it currently stands. Therefore, we invite you to submit a revised version of the manuscript that addresses the points raised during the review process.

Please, check the last recommendations from the reviewer and try to explain on them point by point.

Please submit your revised manuscript by Dec 27 2020 11:59PM. If you will need more time than this to complete your revisions, please reply to this message or contact the journal office at plosone@plos.org. When you're ready to submit your revision, log on to https://www.editorialmanager.com/pone/ and select the 'Submissions Needing Revision' folder to locate your manuscript file.

Please include the following items when submitting your revised manuscript:

- A rebuttal letter that responds to each point raised by the academic editor and reviewer(s). You should upload this letter as a separate file labeled 'Response to Reviewers'.
- A marked-up copy of your manuscript that highlights changes made to the original version. You should upload this as a separate file labeled 'Revised Manuscript with Track Changes'.
- An unmarked version of your revised paper without tracked changes. You should upload this as a separate file labeled 'Manuscript'.

If you would like to make changes to your financial disclosure, please include your updated statement in your cover letter. Guidelines for resubmitting your figure files are available below the reviewer comments at the end of this letter.

If applicable, we recommend that you deposit your laboratory protocols in protocols.io to enhance the reproducibility of your results. Protocols.io assigns your protocol its own identifier (DOI) so that it can be cited independently in the future. For instructions see: http://journals.plos.org/plosone/s/submission-guidelines#loc-laboratory-protocols

We look forward to receiving your revised manuscript.

Kind regards,

Javier S Castresana

Academic Editor

PLOS ONE

[Note: HTML markup is below. Please do not edit.]

Reviewers' comments:

Reviewer's Responses to Questions

**Comments to the Author**

1. If the authors have adequately addressed your comments raised in a previous round of review and you feel that this manuscript is now acceptable for publication, you may indicate that here to bypass the “Comments to the Author” section, enter your conflict of interest statement in the “Confidential to Editor” section, and submit your "Accept" recommendation.

Reviewer #2: All comments have been addressed

**********

2. Is the manuscript technically sound, and do the data support the conclusions?

The manuscript must describe a technically sound piece of scientific research with data that supports the conclusions. Experiments must have been conducted rigorously, with appropriate controls, replication, and sample sizes. The conclusions must be drawn appropriately based on the data presented. 

Reviewer #2: Yes

**********

3. Has the statistical analysis been performed appropriately and rigorously? 

Reviewer #2: Yes

**********

4. Have the authors made all data underlying the findings in their manuscript fully available?

The PLOS Data policy requires authors to make all data underlying the findings described in their manuscript fully available without restriction, with rare exception (please refer to the Data Availability Statement in the manuscript PDF file). The data should be provided as part of the manuscript or its supporting information, or deposited to a public repository. For example, in addition to summary statistics, the data points behind means, medians and variance measures should be available. If there are restrictions on publicly sharing data—e.g. participant privacy or use of data from a third party—those must be specified.

Reviewer #2: Yes

**********

5. Is the manuscript presented in an intelligible fashion and written in standard English?

PLOS ONE does not copyedit accepted manuscripts, so the language in submitted articles must be clear, correct, and unambiguous. Any typographical or grammatical errors should be corrected at revision, so please note any specific errors here.

Reviewer #2: Yes

**********

6. Review Comments to the Author

Please use the space provided to explain your answers to the questions above. You may also include additional comments for the author, including concerns about dual publication, research ethics, or publication ethics. (Please upload your review as an attachment if it exceeds 20,000 characters)

Reviewer #2: The aim of the present study was to reveal the functional role and significance of ALCAM expression in Medulloblastoma (MB). The authors provide new information of ALCAM protein levels analysed by IHC in a MB FFPE cohort conformed by 45 patient and performed an in silico correlation between ALCAM gene expression and the MB molecular and histological subtypes as well as patient age’s in a large MB cohort from the R2 genomics Platform (Cavalli et al. Cancer Cell 2017). In the second part of the study, the authors carried out several functional in vitro and in vivo assays by silencing ALCAM using RNA interference in order to unveil the function of ALCAM in MB.

Reviewer Concerns:

There has been an improvement in the manuscript compared to the previous version. The authors have ensured that they did not define ALCAM as a WNT-related biomarker and the Results are now presented as an interesting correlation between ALCAM expression and the MB-WNT subgroup. The article is not as a biomarker study but an attractive exploration of the MB underlying biology and unveils an interesting role of ALCAM in MB-WNT subgroup. However, some points must be clarified.

Minor Comments:

- Authors need to clarify the finality and the description of the ROC curve analysis. It is not clear if the ROC curve analysis is meant to select ALCAM IHC positivity cut-point neither how many MB samples are used or their subgroup. Authors must remove the word “diagnosis” from the manuscript in line 325 “To evaluate the reliability of the WNT subgroup diagnosis of MB using ALCAM” as it is misleading.

**********

7. PLOS authors have the option to publish the peer review history of their article (what does this mean?). If published, this will include your full peer review and any attached files.

If you choose “no”, your identity will remain anonymous but your review may still be made public.

**Do you want your identity to be public for this peer review?** For information about this choice, including consent withdrawal, please see our Privacy Policy.

Reviewer #2: No

[NOTE: If reviewer comments were submitted as an attachment file, they will be attached to this email and accessible via the submission site. Please log into your account, locate the manuscript record, and check for the action link "View Attachments". If this link does not appear, there are no attachment files.]

While revising your submission, please upload your figure files to the Preflight Analysis and Conversion Engine (PACE) digital diagnostic tool, https://pacev2.apexcovantage.com/. PACE helps ensure that figures meet PLOS requirements. To use PACE, you must first register as a user. Registration is free. Then, login and navigate to the UPLOAD tab, where you will find detailed instructions on how to use the tool. If you encounter any issues or have any questions when using PACE, please email PLOS at figures@plos.org. Please note that Supporting Information files do not need this step.

Attachment Submitted filename: R2_D-19-17600_20201106.pdf Click here for additional data file.

# Author comment: Author response to Decision Letter 2

**DOI:** 10.1371/journal.pone.0243272.r006

18 Nov 2020

Thank you for your valuable comment. As pointed out, we have removed the word “diagnosis” from the sentence to avoid confusion. As suggested, we have modified the description of the ROC curve analysis and added information on the number of MB cases and the subgroup. In the Methods section of the manuscript, the description of the ROC curve analysis was modified and moved from the sub-section “statistical analysis” to “Immunohistochemistry”.

Attachment Submitted filename: Response to Reviewers.docx Click here for additional data file.

# Editor report: Decision Letter 3

**DOI:** 10.1371/journal.pone.0243272.r007

**Authors:** Javier S Castresana

19 Nov 2020

Activated leukocyte cell adhesion molecule expression correlates with the WNT subgroup in medulloblastoma and is involved in regulating tumor cell proliferation and invasion

PONE-D-19-17600R3

Dear Dr. Kijima,

We’re pleased to inform you that your manuscript has been judged scientifically suitable for publication and will be formally accepted for publication once it meets all outstanding technical requirements.

Within one week, you’ll receive an e-mail detailing the required amendments. When these have been addressed, you’ll receive a formal acceptance letter and your manuscript will be scheduled for publication.

An invoice for payment will follow shortly after the formal acceptance. To ensure an efficient process, please log into Editorial Manager at http://www.editorialmanager.com/pone/, click the 'Update My Information' link at the top of the page, and double check that your user information is up-to-date. If you have any billing related questions, please contact our Author Billing department directly at authorbilling@plos.org.

If your institution or institutions have a press office, please notify them about your upcoming paper to help maximize its impact. If they’ll be preparing press materials, please inform our press team as soon as possible -- no later than 48 hours after receiving the formal acceptance. Your manuscript will remain under strict press embargo until 2 pm Eastern Time on the date of publication. For more information, please contact onepress@plos.org.

Kind regards,

Javier S Castresana

Academic Editor

PLOS ONE

Additional Editor Comments (optional):

Reviewers' comments:

# Editor report: Acceptance letter

**DOI:** 10.1371/journal.pone.0243272.r008

**Authors:** Javier S Castresana

23 Nov 2020

PONE-D-19-17600R3 

Activated leukocyte cell adhesion molecule expression correlates with the WNT subgroup in medulloblastoma and is involved in regulating tumor cell proliferation and invasion 

Dear Dr. Kijima:

I'm pleased to inform you that your manuscript has been deemed suitable for publication in PLOS ONE. Congratulations! Your manuscript is now with our production department. 

If your institution or institutions have a press office, please let them know about your upcoming paper now to help maximize its impact. If they'll be preparing press materials, please inform our press team within the next 48 hours. Your manuscript will remain under strict press embargo until 2 pm Eastern Time on the date of publication. For more information please contact onepress@plos.org.

If we can help with anything else, please email us at plosone@plos.org. 

Thank you for submitting your work to PLOS ONE and supporting open access. 

Kind regards, 

PLOS ONE Editorial Office Staff

on behalf of

Prof. Javier S Castresana 

Academic Editor

PLOS ONE
